# New Hydrolyzable Tannin with Potent Antioxidant and *α*-Glucosidase Inhibitory Activity from Black Tea Produced from *Camellia taliensis*

**DOI:** 10.3390/foods12132512

**Published:** 2023-06-28

**Authors:** Min Chen, Na Li, Hong-Tao Zhu, Man Zhang, Zhao-Hong Duan, Dong Wang, Chong-Ren Yang, Ying-Jun Zhang

**Affiliations:** 1 State Key Laboratory of Phytochemistry and Plant Resources in West China, Kunming Institute of Botany, Chinese Academy of Sciences, Kunming 650201, Chinalina1@mail.kib.ac.cn (N.L.);; 2 University of Chinese Academy of Sciences, Beijing 100049, China; 3 Zhao-Hong Gu-Shu Training Center, Lincang 675911, China

**Keywords:** *Camellia taliensis*, black tea, hydrolyzable tannins, antioxidant activity, *α*-glucosidase inhibitory activity

## Abstract

*Camellia taliensis* (W. W. Smith) Melchior, belonging to the genus *Camellia* sect. *Thea*., is mainly distributed from northern Myanmar to western and southwestern Yunnan province of China, and its leaves have been used to make various teas by the locals of its growing regions. The chemical constituents of *C. taliensis* are significantly related to those of cultivated tea plants, *C. sinensis* and *C. sinensis* var. *assamica*. The HPLC-ESI-MS analysis of black tea prepared from the leaves of *C. taliensis* showed a rich existence of polyphenols. Further comprehensive chemical study led to the separation and recognition of 32 compounds (**1**–**32**), including one new hydrolyzable tannin, 1-*O*-galloyl-4,6-tetrahydroxydibenzofurandicarboxyl-*β*-D-glucopyranose (**1**), and one new natural product (**24**). The known compounds referred to seven hydrolyzable tannins (**2**–**8**), 10 flavonols and glycosides (**9**–**18**), and 14 simple phenolics (**19**–**32**). Their structures were elucidated by comprehensive spectroscopic analyses. Among them, 20 compounds (**2**, **3**, **6**, **7**, **8**, **15**, **17**, **18**, **20**–**22**, **24**–**32**) were isolated from black tea for the first time. Most isolates displayed obvious antioxidant activities on DPPH and ABTS^+^ assays, and the hydrolyzable tannins **1**, **3**–**5**, **7,** and **8** exhibited stronger inhibitory activities on *α*-glycosidase than quercetin and acarbose (IC_50_ = 5.75 and 223.30 μM, respectively), with IC_50_ values ranging from 0.67 to 2.01 μM.

## 1. Introduction

Black tea, a fully fermented tea, is usually prepared from the fresh leaves and/or buds of *Camellia sinensis* or *C. sinensis* var. *assamica*, through catalyzed oxidation by polyphenol oxidase (PPO) contained in the fresh tea leaves. Due to its fragrant aroma and various positive health effects, e.g., antioxidant, anti-hyperlipidemia, anti-hyperglycemic, and anti-microbial activities [1,2,3,4,5], it is increasingly attracting attention from both consumers and researchers as well as becoming a mainstream commodity in the international tea trade, accounting for about 80% of the global tea trade [6]. To date, nearly 100 compounds were separated from black tea, such as theaflavins [7,8], catechins and their derivatives [4,9], flavonols and related glycosides [3,10], flavoalkaloids [4,11], minorly hydrolyzable tannins [9], and alkaloids [12].

*C. taliensis* (W. W. Smith) Melchior is the most extensively distributed wild tea tree in the genus *Camellia* sect. *Thea*. [13,14], and primarily distributed from the north of Myanmar to the west and southwest of Yunnan province, China, with a scattered distribution along the Ailao Mountain and Lancang (Mekong) and Nujiang (Salween) river basins of China [14,15]. The chemical study of *C. taliensis* may contribute to increasing its popularity and further promote the economic development of its growing regions. Our previous study showed that *C. taliensis* and cultivated tea plants (*C. sinensis* and *C. sinensis* var. *assamica*) were alike in terms of their chemical compositions, all containing abundant flavan-3-ols and caffeine, but a slightly lower content in the case of *C. taliensis*. In addition, the rich hydrolyzable tannins is one of the characteristics of *C. taliensis*, among which 1,2-di-*O*-galloyl-4,6-*O*-(*S*)-hexahydroxydiphenoyl-*β*-D-glucopyranose was considered to be a marker component as its content in dried leaves reached up to 2.44% [13]. As the earliest and mostly used wild tea tree, *C. taliensis* has been used as a raw material to manufacture various teas, e.g., green tea, raw Pu-er tea, and black tea by the locals of its growing regions [16,17].

Our phytochemical study of green tea prepared from the leaves of *C. taliensis* revealed 33 compounds, including 12 hydrolyzable tannins, 7 flavan-3-ol derivatives, 5 flavonols and glycosides, 8 simple phenolics, and caffeine. Most of them had significant antioxidant activities [2,18]. Moreover, 91 volatile constituents with antioxidant activities were also discovered in its unprocessed leaves and green tea [16]. As a part of our ongoing chemical study of tea and its original plant, black tea produced from the leaves of *C. taliensis*, gathered from the Fengqing County of Yunnan province, China, was chemically investigated. The HPLC-MS combined with electrospray ionization (HPLC-ESI-MS) analysis indicated the rich presence of polyphenols. Further comprehensive chemical study on its EtOAc extract resulted in the separation and recognition of 32 phenolic compounds, which were 8 hydrolyzable tannins (**1**–**8**) with one new tannin (**1**), 10 flavonols and its glycosides (**9**–**18**), and 14 simple phenolics (**19**–**32**) with one new natural product (**24**). The antioxidant activity on the DPPH and ABTS^+^ assays of most isolates and *α*-glucosidase inhibitory activity of all hydrolyzable tannins were tested. Herein, we report the study.

## 2. Materials and Methods

### 2.1. General Procedure

UV spectra were recorded using a Shimadzu UV2401A spectrophotometer (Shimadzu Co., Kyoto, Japan) with methanol as a solvent. The optical rotations in methanol were detected with a P-1020 polarimeter (JASCO, Tokyo, Japan). A chirascan V100-Applied Photophysics spectrometer (Applied Photophysics Ltd., Leatherhead, Surrey, UK) was applied to collect the electronic circular dichroism data. Bruker NMR (600 MHz and 500 MHz) spectrometers (Bruker Co., Karlsruhe, Germany) were used to record all NMR spectra in methanol-*d*_4_ (CD_3_OD) using solvent peaks as references. Chemical shifts were denoted by *δ* (ppm) and coupling constants were indicated as *J* (Hz). ESIMS and HRESIMS data were recorded on a Shimadzu LCMS-2020 (Shimadzu Corporation, Tokyo, Japan). LC-MS analysis was monitored on Agilent 1290 (Agilent Technologies Inc., Santa Clara, USA) equipped with COSMOSIL C-18 column (4.6 × 250 mm inner diameter, 5 μm) with DAD detection. Semi-HPLC was performed on a Hanbon HPLC (Hanbon Sci. & Tech., Huai’an, China) and equipped with a Thermo Hypersil GOLD aQ column (10 × 250 mm inner diameter, 5 μm).

### 2.2. Chemicals and Reagents

Diaion HP20SS (63−150 µm) and MCI-gel CHP20P (75−100 μm) (Mitsubishi Chemical Co., Tokyo, Japan), Sephadex LH-20 (25−100 μm) and TSK gel Toyopearl HW-40F (37−70 μm) (GE Healthcare Bio-Science AB, Uppsala, Sweden), and RP-18 (40−60 μm, Merck, Darmstadt, Germany) were used as the padding of column chromatography (CC). Thin-layer chromatography (TLC) was carried out using precasted silica gel F254 plates (Qingdao Haiyang Chemical Co., Ltd. Qingdao, China) using methylbenzene:ethyl formate:formic acid (Shanghai Titan Scientific Co., Ltd., Shanghai, China) (1:7:2, 1:7:1, 2:7:1, 3:6:1, 4:5:1, *v*/*v*/*v*) as the eluents. UV radiation was used to locate the spots by soaking with a sulfuric acid:ethanol (1:9, *v*/*v*) solution followed by heating. The deionized water and redistilled organic solvents such as acetone, chloroform (CHCl_3_), methanol (MeOH), ethyl acetate (EtOAc), ethanol (EtOH), and *n*-butanol (*n*-BuOH) were used for column chromatography. The chromatographic-grade acetonitrile (MeCN), formic acid (HCOOH), and deionized water (H_2_O) were used for HPLC.

### 2.3. Materials

Black tea from *C. taliensis* was gathered in Dasi township, Fengqing County, Yunnan Province, China, in 2021. The specimen (KIB-Z-2107B17) was stored in the State Key Laboratory of Phytochemistry and Plant Resources in West China, Kunming Institute of Botany (KIB), Chinese Academy of Sciences (CAS).

### 2.4. HPLC and LC-MS Analysis

The fine powder (4.0 g) of black tea from *C. taliensis* was ultrasonically extracted twice (20 min each time) during 12 h with 70% MeOH (150 mL) at room temperature. The extract was first dried down to produce a crude residue, which was then dissolved with water and redistributed using CHCl_3_ to weaken the interference from caffeine. For further HPLC and LC-MS analyses, the aqueous fraction was filtered over a 0.22 μm nylon membrane.

Then, 20 μL sample solution was applied for the HPLC analysis conducted with an Agilent Zorbax SB C-18 column (4.6 × 250 mm inner diameter, 5 μm), with a gradient elution of 4–40% (45 min) MeOH−H_2_O (containing 1.5‰ HCOOH) solution (1 mL/min) as a mobile phase. The chromatogram was collected at 240, 254, 280, 300, 330, and 350 nm. The temperature of the column was kept at 30 °C. The electrospray ionization (ESI) with two modes (negative and positive ionization), working with a full-scan mode (100−1500 *m*/*z*), was applied to perform MS analysis. The following operation parameters were applied: the ion spray voltage, 4 kV; temperature, 400 °C.

### 2.5. Extraction and Isolation

Black tea from *C. taliensis* (10.2 kg) was extracted using 60% aqueous acetone (40 L × 4, 1 week for each) at room temperature. After recycling acetone under reduced pressure, the yielded crude extract was dissolved with water and subsequently redistributed using CHCl_3_, EtOAc, and *n*-BuOH. The EtOAc fraction (350 g) was further fractionated by Sephadex LH-20 CC eluted with aqueous MeOH (0–100%) to give six fractions (Fr. 1–Fr. 6).

Fr. 2 (32.2 g) was treated with MCI-gel CHP20P CC (42 × 6 cm) eluted with aqueous MeOH (0−100%) to give compounds **19** (5.09 g) and **20** (0.39 g) and another 15 subfractions (Fr. 2-1–Fr. 2-15). Compound **3** (10.2 mg) was obtained from Fr. 2-1 (286.6 mg) by repeated CC over RP-18 and Toyopearl HW-40F eluted with aqueous MeOH (0–100%) and semi-HPLC (aqueous MeCN, 7%). Compound **30** (15.3 mg) and compounds **6** (11.9 mg), **21** (3.8 mg), **25** (12.2 mg), **28** (9.8 mg) were achieved from Fr. 2-3 (1.96 g) and Fr. 2-4 (1.58 g), respectively, via Sephadex LH-20 CC (aqueous MeOH, 0–100%) and further semi-HPLC (aqueous MeCN). Fr. 2-5 (1.28 g) was manipulated with Toyopearl HW-40F CC using aqueous MeOH (0–100%) as an eluent, combined with semi-HPLC (aqueous MeCN) to give **2** (1.9 mg), **4** (36.3 mg), **22** (2.0 mg), **23** (33.4 mg), **26** (10.6 mg), **27** (7.6 mg). Multiple CC over Toyopearl HW-40F, RP-18, and Sephadex LH-20 using aqueous MeOH (0−100%) as eluent, followed with semi-HPLC (aqueous MeCN) afforded **9** (15.1 mg), **11** (1.4 mg), **12** (43.0 mg), **13** (13.3 mg), **17** (10.6 mg), **18** (8.7 mg), **29** (21.5 mg) from Fr. 2-7 (9.43 g), and **14** (4.5 mg) and **15** (4.5 mg) from Fr. 2-8 (4.67 g), respectively. Fr. 2-9 (1.80 g) and Fr. 2-10 (0.99 g) were manipulated using Toyopearl HW-40F CC eluted with aqueous MeOH (0−100%), followed by semi-HPLC (aqueous MeCN with 1.5‰ HCOOH, 30% and 34%) to give **24** (13.6 mg) and **16** (9.4 mg), respectively.

Fr. 3 (121.2 g) was treated using Diaion HP20SS CC (60 × 7 cm), with aqueous MeOH (0−100%) as eluent, to afford 15 subfractions (Fr. 3-1–Fr. 3-15). Fr. 3-7 (16.93 g) was manipulated with repeated CC over Toyopearl HW-40F, RP-18, MCI-gel CHP20P, and Sephadex LH-20 eluted with a gradient system of aqueous MeOH (0–100%), followed with semi-HPLC (aqueous MeCN) to give **1** (8.4 mg), **5** (3.4 mg), **7** (3.5 mg), **8** (13.5 mg), and **31** (7.3 mg). Fr. 3-11 (3.29 g) was processed with multiple CC over Toyopearl HW-40F and Sephadex LH-20 eluted with aqueous MeOH (0–100%), followed with semi-HPLC (aqueous MeCN with 1.5‰ HCOOH, 27%) to give **10** (3.6 mg). Compound **32** (3.3 mg) was gained from Fr. 3-12 (6.70 g) through multiple CC over Toyopearl HW-40F and Sephadex LH-20 with aqueous MeOH (0–100%) as eluent, followed by semi-HPLC (aqueous MeCN, 35%).

### 2.6. Compound ***1***

Yellow-brown amorphous powder; ${\left[\alpha \right]}_{D}^{20}$+34.4 (0.10, MeOH); ESI-MS: *m*/*z* 615 [M − H]^−^; HRESI-MS: *m*/*z* 615.0625 [M − H]^−^ (calculated for C_27_H_19_O_17_, 615.0628); UV *λ*_max_ (MeOH) (log *ε*): 276 (4.35), 222 (4.51) nm; ^1^H NMR (500 MHz, CD_3_OD): *δ*_H_ 7.20 (s, H-3″), 7.12 (s, 2H, H-2′, 6′), 7.05 (s, H-3‴), 5.73 (d, *J* = 8.2 Hz, H-1), 5.00 (m, H-4), 4.78 (m, H-6a), 4.01 (m, H-6b), 3.98 (m, H-5), 3.86 (t, *J* = 9.4 Hz, H-3), 3.65 (dd, *J* = 9.4, 8.2 Hz, H-2). ^13^C NMR (125 MHz, CD_3_OD): *δ*_C_ 171.5 (C-7‴), 170.0 (C-7″), 166.8 (C-7′), 147.7 (C-6‴), 147.6 (C-6″), 146.6 (C-3′, 5′), 145.5 (C-4‴), 145.2 (C-4″), 140.6 (C-4′), 136.1 (C-5″), 134.0 (C-5‴), 120.4 (C-1′), 119.8 (C-2‴), 117.0 (C-2″), 116.2 (C-3″), 115.9 (C-1″), 115.4 (C-1‴), 112.1 (C-3‴), 110.6 (C-2′, 6′), 95.7 (C-1), 76.2 (C-4), 74.9 (C-3), 74.2 (C-2), 71.7 (C-5), 67.6 (C-6).

### 2.7. Antioxidant Activity

Assays for DPPH and ABTS^+^ were carried out using the previous method with some modifications [19]. The DPPH assay was conducted as follows: the tested compound (final concentration: 0.2–1000 μM in EtOH, 100 μL) and DPPH solution (200 μM in EtOH, 100 μL) were sequentially added to 96-well plates at room temperature, then hid from the light for 15 min. The blank control and positive control (ascorbic acid) were set simultaneously. OD values at 517 nm were detected with a microplate reader. ABTS^+^ assay was carried out as follows: firstly, the ABTS^+^ solution was prepared using the procedure in our previous paper [19]. Then, the tested compound (final concentration: 0.2–1000 μM in EtOH, 10 μL) and ABTS^+^ solution (200 μL) were added to 96-well plates at room temperature in an orderly manner, and then hided from light for 6–8 min. The blank control and positive control (trolox) were set synchronously. OD values at 405 nm were then detected with a microplate reader. All the reactions were set up three replicates. The formula for calculating the scavenging percentage is as follows: inhibition percentages (%) = (OD_blank_ − OD_test_)/OD_blank_ × 100%, and IC_50_ values were computed based on the Reed–Muench method [20].

### 2.8. α-Glucosidase Inhibitory Activity

As recorded in our prior paper [4], 4-nitrophenol-*α*-D-glucopyranoside (PNPG) was used as the zymolyte for the screening of enzyme inhibitors. Initially, 96-well plates with the following ingredients were added in a proper order: testing compound (50 μM), *α*-glucosidase solution (0.025 U/mL), buffer, and the zymolyte (1 mM), and kept at 37 °C for 50 min. Quercetin and acarbose were used as the positive control. All the reactions were set up with three replicates. OD values at 405 nm were recorded with a microplate reader. The formula for calculating the scavenging percentage is as follows: inhibition percentages (%) = (OD_blank_ − OD_test_)/OD_blank_ × 100%, and IC_50_ values were computed based on the Reed–Muench method [20].

## 3. Results and Discussion

### 3.1. HPLC and LC-MS Analysis

HPLC-ESI-MS analysis of the extract of black tea from *C. taliensis* using 70% aqueous MeOH revealed a total of 33 chemical constituents, including mainly polyphenols, e.g., 5 catechins, 3 theaflavins, 4 hydrolyzable tannins, 6 simple phenolics, 13 flavonols and their glycosides and coumarin, as well as theanine (Table 1), on the basis of their quasi-molecular ions, fragment ions, UV absorption, and retention times (*t*_R_), combined with standards obtained in our prior studies. Half of them were also isolated in further chemical study.

**Figure 1:**
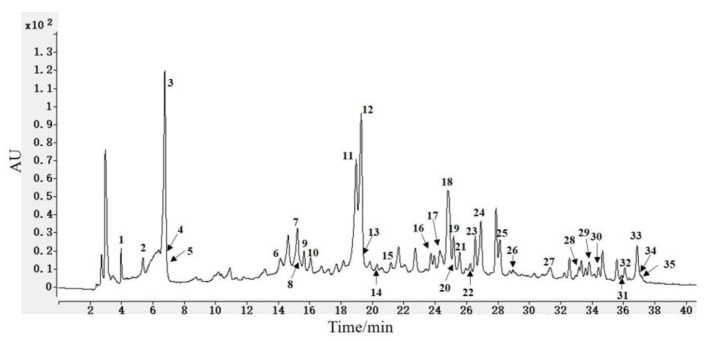
HPLC chromatogram for the extract of black tea from *C. taliensis* using 70% MeOH.

Catechins were found at peaks 8, 11, 12, 19, and 26 in Figure 1. Peak 8 was recognized as (+)-catechin through the [M − H]^−^ quasi-molecular ion at *m*/*z* 289, *t*_R_ (15.55 min) and UV absorption (*λ*_max_ 212, 274 nm), combined with those of the reference substance. Thus, (−)-epicatechin, (−)-epigallocatechin-3-*O*-gallate, (−)-epicatechin-3-*O*-gallate, and (−)-epiafzelechin 3-*O*-gallate were discovered as peaks 11 (*t*_R_ 18.71 min; *λ*_max_ 211, 274 nm), 12 (*t*_R_ 19.22 min; *λ*_max_ 209, 271 nm), 19 (*t*_R_ 24.96 min; *λ*_max_ 209, 273 nm) and 26 (*t*_R_ 29.04 min; *λ*_max_ 210, 278 nm) by the same token, respectively.

Peaks 32, 33, and 34 in Figure 1 were speculated to be theaflavins. Peak 33 was identified as theaflavin-3,3′-digallate according to fragment ions at *m*/*z* 715 [M − galloyl]^−^, 697 [M − H − gallic acid]^−^, and compared the *t*_R_ (36.94 min) and UV absorptions (*λ*_max_ 210, 276 nm) with those of the reference substance. In a similar way, peaks 32 (*m*/*z* 563 [M − galloyl]^−^; *t*_R_ 36.15 min; *λ*_max_ 210, 272 nm) and 34 (*m*/*z* 563 [M − galloyl]^−^; *t*_R_ 37.07 min; *λ*_max_ 210, 277 nm) were confirmed as theaflavin-3-gallate and theaflavin-3′-gallate, respectively.

Peaks 2, 6, 9, and 14 in Figure 1 were conjectured to be hydrolyzable tannins. Due to the fragment ions (*m*/*z* 331 [M − H]^−^, 169 [M − H − glucosyl]^−^) and *t*_R_ (5.43 min), peak 2 was identified as *β*-glucogallin. Similarly, peaks 6 (*m*/*z* 313 [M − H − gallic acid]^−^; *t*_R_ 14.17 min; *λ*_max_ 230, 266, 297 nm), 9 (*m*/*z* 463 [M − H − gallic acid]^−^, 301 [463 − glucosyl]^−^; *t*_R_ 15.70 min; *λ*_max_ 227, 270 nm) and 14 (*m*/*z* 483 [M − galloyl]^−^, 169 [gallic acid − H]^−^; *t*_R_ 20.36 min; *λ*_max_ 223, 277 nm) were recognized as 1,6-di-*O*-galloyl-*β*-D-glucopyranose (**6**), strictinin (**4**) and 1,4,6-tri-*O*-galloyl-*β*-D-glucopyranose (**8**), respectively, and these were obtained during further chemical isolation.

Peaks 3, 4, 5, 7, 10, and 13 in Figure 1 were presumed to be simple phenolic compounds. Peaks 4 (*m*/*z* 125 [M − H]^−^; *t*_R_ 6.82 min) and 5 (*m*/*z* 343 [M − H]^−^; *t*_R_ 6.82 min) were identified as pyrogallol and theogallin, respectively. Similarly, gallic acid (**19**), methyl gallate (**23**), chlorogenic acid (**25**), and 5-*O*-(*E*)-*p*-coumaroylquinic acid (**26**) were determined at peaks 3 (*m*/*z* 169 [M − H]^−^, 125 [M − COO]^−^; *t*_R_ 6.82 min; *λ*_max_ 227, 275 nm), 7 (*m*/*z* 169 [M − CH_3_]^−^, 125 [M − COOCH_3_]^−^; *t*_R_ 15.31 min; *λ*_max_ 227, 270 nm), 10 (*m*/*z* 191 [quinic acid − H]^−^, 179[caffeic acid − H]^−^; *t*_R_ 16.12 min; *λ*_max_ 218, 328 nm), and 13 (*m*/*z* 191 [quinic acid − H]^−^, 163 [coumaric acid − H]^−^; *t*_R_ 19.34 min; *λ*_max_ 228, 307 nm), respectively. All of them were obtained during further chemical isolation.

Peaks 16, 18, 20, 21, 22, 23, 24, 25, 27, 28, 30, 31, and 35 in Figure 1 were inferred to be flavonols and their glycosides. Peak 16 was identified as rutin (**13**) by the fragment ions (*m*/*z* 308 [glucosyl + rhamnosyl]^−^, 301 [quercetin]^−^), *t*_R_ (23.79 min) and UV absorption (*λ*_max_ 216, 257, 354 nm). Likewise, peaks 18 (*m*/*z* 301 [quercetin]^−^; *t*_R_ 24.85 min; *λ*_max_ 210, 257, 354 nm), 21 (*m*/*z* 447 [M − rhamnosyl]^−^, 285 [447 − glucosyl]^−^; *t*_R_ 25.63 min, *λ*_max_ 260, 302, 362 nm), 22 (*m*/*z* 302 [quercetin]^−^; *t*_R_ 26.31 min), 23 (*m*/*z* 593 [M − H]^−^; *t*_R_ 26.62 min; *λ*_max_ 210, 257, 350 nm), 24 (*m*/*z* 285 [M − H − glucosyl]^−^; *t*_R_ 27.16 min; *λ*_max_ 226, 264, 348 nm), 25 (*m*/*z* 285 [M − H − galactosyl]^−^; *t*_R_ 28.21 min; *λ*_max_ 210, 266, 347 nm), 27 (*m*/*z* 285 [M − H − rhamnosyl]^−^; *t*_R_ 31.45 min; *λ*_max_ 210, 264, 343 nm), 31 (*m*/*z* 447 [M − 2H − coumaroyl]^−^, 285 [447 − glucosyl]^−^; *t*_R_ 35.95 min; *λ*_max_ 210, 266, 315 nm) and 35 (*m*/*z* 301 [M − H]^−^; *t*_R_ 37.24 min; *λ*_max_ 210, 256, 371 nm) were recognized as quercetin 3-*O*-*β*-D-glucopyranoside (**11**), kaempferol 3-*O*-rutinoside (**12**), quercetin 3-(2″-*β*-D-glucosy-l)-*α*-L-rhamnopyranoside (**17**), kaempferol 3-(2″-*β*-D-glucosyl)-*α*-L-rhamnopyranoside (**18**), kaempherol 3-*O*-*β*-D-glucopyranoside (**9**), kaempherol 3-*O*-*β*-D-galacopyranoside (**14**), kaempherol 3-*O*-*α*-L-rhamnopyranoside (**15**), kaempferol 3-*O*-(6″-*trans*-*p*-coumaroy-l)-*β*-D-glucopyranoside (**10**), and quercetin (**16**), respectively, and they were also isolated from further chemical study. In addition, peaks 20 (*m*/*z* 463 [M − H]^−^; *t*_R_ 25.25 min), 28 (*m*/*z* 755 [M − H − rhamnosyl]^−^, 447 [M − H − glucosyl − coumaroyl]^−^; *t*_R_ 33.19 min), 30 (*m*/*z* 739 [M − H − rhamnosyl]^−^, 577 [739 − glucosyl]^−^, 431 [577 − H − coumaroyl]^−^, 285 [431 − rhamnosyl]^−^; *t*_R_ 34.28 min), were speculated to be quercetin 3-*O*-*β*-D-galactopyranoside, quercetin-3-*O*-coumaroyl-rhamnosyl-glucosyl-rhamnopyranoside, and kaempferol-3-*O*-coumaroyl-rhamnosyl-glucosyl-rhamnopyranoside, respectively.

In addition, two other types of compounds were also identified. Compared the quasi-molecular ion of *m*/*z* 173 [M − H]^−^ and fragment ions (*m*/*z* 127, 87, 45) with those of the standard sample, peak 1 was inferred to be theanine. Similarly, peak 15 (*m*/*z* 177 [M − H]^−^, 132, 109, 89, 65; *t*_R_ 21.24 min; *λ*_max_ 210, 255, 328 nm) was identified as 5,7-dihydroxycoumarin.

### 3.2. Identification of Compounds ***1**–**32***

**Figure 2:**
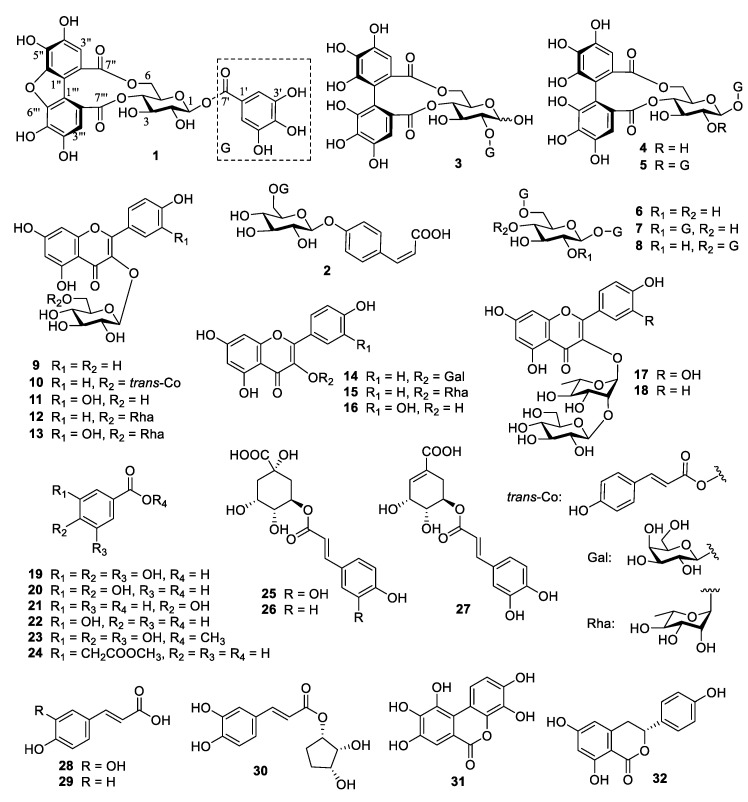
Compounds **1**–**32** isolated from black tea from *C. taliensis*.

The extract of black tea from *C*. *taliensis* using 60% aqueous acetone was dissolved with water and successively redistributed with CHCl_3_, EtOAc, and *n*-BuOH. The EtOAc extract was further isolated by various CC on Diaion HP20SS, RP-18, Toyopearl HW-40F, MCI-gel CHP20P, and Sephadex LH-20, to yield 32 compounds, including one undescribed hydrolyzable tannin (**1**) and one new natural product (**24**). The compounds, which had were in advance, were recognized as seven hydrolyzable tannins (**2**−**8**) including 4-*O*-(6′-*O*-galloyl-*β*-D-glucopyranosyl)-*cis*-*p*-coumaric acid (**2**) [21], 2-*O*-galloyl-4,6-*O*-(*S*)-hexahydroxydiphenoyl-*β*-D-glucopyranose (**3**) [22], 1-*O*-galloyl-4,6-*O*-(*S*)-hexahydroxydiphenoyl-*β*-D-glucopyranose (**4**) [23], 1,2-di-*O*-galloyl-4,6-*O*-(*S*)-hexahydroxydiphenoyl-*β*-D-glucopyranose (**5**) [24], 1,6-di-*O*-galloyl-*β*-D-glucopyranose (**6**) [25], 1,2,6-tri-*O*-galloy-*β*-D-glucopyranose (**7**) [26], and 1,4,6-tri-*O*-galloyl-*β*-D-glucopyranose (**8**) [27], 10 flavonol and its glycosides (**9**−**18**) including kaempferol 3-*O*-*β*-D-glucopyranoside (**9**) [28], kaempferol 3-*O*-(6″-*trans*-*p*-coumaroyl)-*β*-D-glucopyranoside (**10**) [29], quercetin 3-*O*-*β*-D-glucopyranoside (**11**) [30], kaempferol 3-*O*-rutinoside (**12**) [23], rutin (**13**) [31], kaempferol 3-*O*-*β*-D-galactopyranoside (**14**) [32], kaempferol 3-*O*-*α*-L-rhamnopyranoside (**15**) [33], quercetin (**16**) [34], quercetin 3-(2″-*β*-D-glucosyl)-*α*-L-rhamnopyranoside (**17**) [35], and kaempferol 3-(2″-*β*-D-glucosyl)-*α*-L-rhamnopyranoside (**18**) [35], and 14 simple phenolics (**19**−**32**) including gallic acid (**19**) [36], 3,4-dihydroxybenzoic acid (**20**) [37], *p*-hydroxybenzoic acid (**21**) [38], *m*-hydroxybenzoic acid (**22**) [39], methyl gallate (**23**) [40], 3-(2-methoxy-2-oxoethyl) benzoic acid (**24**) [41], chlorogenic acid (**25**) [42], 5-*O*-(*E*)-*p*-coumaroylquinic acid (**26**) [43], 5-*O*-caffeoylshikimic acid (**27**) [44], caffeic acid (**28**) [45], *E*-*p*-hydroxycinnamic acid (**29**) [46], 1-(3′,4′-dihydroxycinnamoyl) cyclopentane-2,3-diol (**30**) [47], 3,4,8,9,10-pentahydroxydibenzo[b,d]pyran-6-one (**31**) [48], and (3*R*)-thunberginol C (**32**) [49], respectively, based on previously reported MS, and NMR spectroscopic data (Figure 2). Before this study, the 20 compounds (**2**, **3**, **6**, **7**, **8**, **15**, **17**, **18**, **20**–**22**, **24**–**32**) were never been separated from black tea.

**Figure 3:**
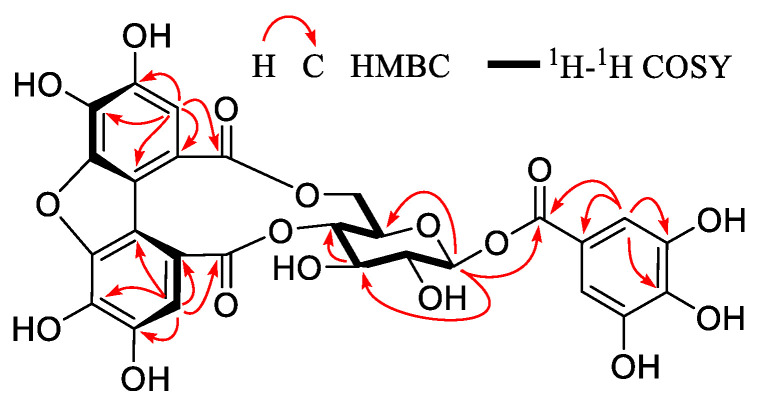
Key 2D NMR correlations of compound **1**.

Compound **1** was obtained as a yellow-brown amorphous powder with ${\left[\alpha \right]}_{D}^{20}$+34.4 (*c* 0.10, MeOH) (Figure S9). Its molecular formula, C_27_H_20_O_17_, was deduced from HRESI-MS at *m*/*z* 615.0625 [M − H]^−^ (calculated for 615.0628) (Figure S7), demonstrating 18 degrees of unsaturation. Detailed analysis of the NMR data revealed that **1** had a great semblable structure to that of strictinin (**4**) (Figures S1 and S2). The existence of one glucosyl (*δ*_C_ 95.7, 76.7, 74.9, 74.2, 71.7, 67.6; *δ*_H_ 5.73, 1H, d, *J* = 8.2 Hz) with C1 form proved by the study on the conformation of ellagitannins (ETs) [50] and the large coupling constant (*J* = 8.2 Hz) of anomeric proton (*δ*_H_ 5.73) [51], one galloyl (*δ*_C_ 146.6 (2C), 140.6, 120.4, 110.6 (2C), 166.8; *δ*_H_ 7.12, 2H, s), and one HHDP-related acyl (*δ*_C_ 147.7, 147.6, 145.5, 145.2, 136.1, 134.0, 119.8, 117.0, 116.2, 115.9, 115.4, 112.1, 171.5, 170.0; *δ*_H_ 7.20, 7.05. each 1H, s) groups were deduced apparently from the ^1^H and ^13^C NMR spectra (Table 2). The HMBC correlation from the anomeric proton (*δ*_H_ 5.73) to galloyl carboxyl carbon (*δ*_C_ 166.8) clarified that the galloyl group was connected at glucosyl C-1. The obvious lower field shift of glucosyl C-6 (*δ*_C_ 67.6), H-4 (*δ*_H_ 5.00), and H-6 (*δ*_H_ a, 4.78; b, 4.01) compared with those of 1-*O*-galloyl-*β*-D-glucopyranose [52], suggested the HHDP-related acyl group should be attached to the glucosyl C-4 and C-6 positions, analogous to those of **4**. However, ^1^H and ^13^C NMR data assignable the HHDP-related acyl group in **1** were very different to those of the HHDP group in **4**, and the molecular formula of **1** was differed from **4** by one H_2_O, indicating that **1** should be a dehydrated derivative of **4**. The HHDP-related acyl group at glucosyl C-6 and C-4 in **1** was determined to be 1,1′-(3,3′,4,4′-tetrahydroxy) dibenzofurandicarboxyl group, compared with the NMR data with those in mallotusinin, the first compound with a 1,1′-(3,3′,4,4′-tetrahydroxy) dibenzofurandicarboxyl group isolated from *Mallotus japonicus* [53]. Other 2D NMR correlations (Figure 3 and Figure S3–S6) confirmed the structure of **1**. Hence, compound **1** was confirmed to be 1-*O*-galloyl-4,6-tetrahydroxydibenzofurandicarboxyl-*β*-D-glucopyranose, as shown in Figure 2.

Hydrolyzable tannins are segmented into two major classes based on their structures: gallotannins (GTs) and ETs. GTs are esters with only a galloyl group bounded to glucose, while ETs are esters with hexahydroxydiphenoyl (HHDP) or its similar acyl groups commonly bounded to glucose [50]. Isolates **6**–**8** were classified as GTs, and **1**, **3**, **4,** and **5** were classified as ETs. ETs are often present as an equilibrium mixture of two isomers with a different configuration at anomeric carbon [50]. For example, compound **3** was isolated as a mixture, due to the chirality of anomeric carbon. It was reported that the original product of the oxidative coupling of two galloyl groups in ETs was the dehydrohexahydroxydiphenoyl (DHHDP) group, which was then reduced to the HHDP group [54], as shown in Figure S10. The DHHDP group in dehydroellagitannins usually comes simultaneously in six-membered and five-membered hemiacetal rings. However, due to the influence of Gibbs free energy, the stability of the six-membered ring structure of DHHDP is better than that of the five-membered ring structure. Meanwhile, the pressure load carried by ester carbonyl carbons and the flexibility of the macrocylic lactone ring in ETs make a difference to the stability of DHHDP [54]. Consequently, the structure, molecule size, and location of acyl group are all related to the structural diversity of ETs, which will affect the stability of DHHDP. A study found that the DHHDP group, created with pyridine in acetonitrile, was disproportionated by redox reaction to yield the 1,1′-(3,3′,4,4′-tetrahydroxy) dibenzofurandicarboxyl group as a reduction product [55]. Thus, compound **1** could be a reduction product, reduced through the two galloyl groups of **4** which were converted into DHHDP by the oxidative coupling reaction, followed by redox disproportionation. 

Urolithins, as natural metabolites of ETs with better gastrointestinal absorption, were reported to have inhibitory effects on the proliferation of prostate and colon cancer cells as well as anti-inflammation activity [56]. It is reported that ETs were hydrolyzed by intestinal bacteria to produce ellagic acid, which were subsequently converted into urolithins. The presence of compound **31** (resembled a urolithin) demonstrated a possible occurrence of the ester hydrolysis of ET acyl groups and further decarboxylation during the fermentation of black tea made from the leaves of *C*. *taliensis*.

### 3.3. Antioxidant Activity

The antioxidant activities through DPPH and ABTS^+^ assays for all of the hydrolyzable tannins (**1**–**8**), flavonol and its glycosides (**9**–**18**), and simple phenolic compounds (**19**–**23**, **25**–**32**) isolated from *C*. *taliensis* black tea, were assessed. The results, shown in Table 3, indicated that the amount of phenolic hydroxyl group was clearly correlated with their antioxidant activities, and the sequence of activity was hydrolyzable tannins > simple phenolics > flavonol and its glycosides. 

All eight hydrolyzable tannins (**1**–**8**) showed a stronger free radical scavenging activities than ascorbic acid and trolox. The sequence of activity for inhibiting the DPPH radical was **8** > **6** > **5** > **4** > **1** > **2** > **7** > **3** > ascorbic acid, and the sequence of activity for inhibiting the ABTS^+^ radical was **5** > **8** > **4** > **6** > **3** > **1** > **7** > **2** > trolox. In hydrolyzable tannins, galloyl group at C-2 of glucose could weaken the radical scavenging effects from the weaker results of **3** and **7** than those of **4** and **8**. 

Part of the flavonol and its glycosides (**11**, **13**, **16**, **17**) showed equivalent effects to the positive control (ascorbic acid and trolox). The sequence of activity for inhibiting the DPPH radical was **13** > **16** > **11** > ascorbic acid > **17**, and the sequence of activity for inhibiting the ABTS^+^ radical was **16** > **13** > **11** > trolox > **17**. The *p*-coumaryl group has a little positive influence on the antioxidant effects of flavonol and its glycosides, which could be found from **10** (6.28%) and **9** (0.97%), respectively. 

Part of the simple phenolic compounds (**19**, **23**, **25**, **28**, **30**, **31**) showed stronger activity than the positive control (ascorbic acid and trolox). The sequence of activity for the inhibiting DPPH radical was **19** > **23** > **31** > **25** > **28** > **30** > ascorbic acid, and the sequence of activity for inhibiting the ABTS^+^ radical was **31** > **23** > **28** > **19** > **25** > **30** > trolox. The *p*-coumaric acid derivatives showed little radical scavenging effects, weaker than caffeic acid derivatives from the order of activity (**25** > **30** > **27** > **26** > **29**), which was consistent with reports in the literature [57]. 

### 3.4. α-Glucosidase Inhibitory Activity

The inhibitory activities of hydrolyzable tannins **1**–**8** and gallic acid (**19**) on *α*-glucosidase were investigated. At a concentration of 50 μM, **1**, **3**–**5**, **7** and **8** with three or more galloyl groups showed a higher inhibition ratio (>50%) on *α*-glucosidase. Their IC_50_ values were further evaluated, and as shown in Table 4, all showed a stronger inhibitory activity than quercetin and acarbose (IC_50_ = 5.75 and 223.30 μM, respectively), with IC_50_ values ranging from 0.67 to 2.01 μM. Their activity order was **5** > **3** > **1** > **4** > **7** > **8** > quercetin > acarbose. Compounds **2** and **6** with one or two galloyl groups showed equivalent inhibitory effects to acarbose on *α*-glucosidase at a concentration of 50 μM, while gallic acid (**19**) showed almost no inhibitory effect. The results revealed that the number of phenolic hydroxyl group plays a positive role on the *α*-glucosidase inhibitory activities of hydrolyzable tannins, probably due to their hydrophobic association with *α*-glucosidase [58].

## 4. Conclusions

*C. taliensis*, with similar chemical constituents to the extensively cultivated tea plants, *C. sinensis* and its variety *assamica*, is the earliest, most used and widely distributed wild tea tree, whose leaves were utilized by the locals of its growing regions to manufacture various types of tea. A comprehensive chemical study of black tea from *C. taliensis* resulted in the separation and recognition of one new hydrolyzable tannin, 1-*O*-galloyl-4,6-tetrahydroxydibenzofurandicarboxyl-*β*-D-glucopyranose (**1**), together with 31 known compounds, comprising seven hydrolyzable tannins (**2**–**8**), 10 flavonols and their glycosides (**9**–**18**), and 14 simple phenolics (**19**–**32**) with **24** as a new natural product. It is noted that 15 compounds (**2**, **3**, **15**, **17**, **18**, **21**, **22**, **24**, **26**–**32**) were obtained from *C*. *taliensis* and 20 compounds (**2**, **3**, **6**, **7**, **8**, **15**, **17**, **18**, **20**–**22**, **24**–**32**) were isolated from black tea for the first time. The isolation of hydrolyzable tannins, especially new ones from fermented tea, is challenging, because hydrolyzable tannins are easily hydrolyzed during fermentation process. Nonetheless, one new hydrolyzable tannin was isolated and identified in the study. As far as we know, the hydrolyzable tannin with 1,1′-(3,3′,4,4′-tetrahydroxy) dibenzofurandicarboxyl group was discovered from tea for the first time. Most of the isolates showed significant antioxidant and *α*-glucosidase inhibitory activities, which were positively correlated with the amount of phenolic hydroxyl group. However, more antioxidant and hypoglycemic activity experiments could be carried out. In short, *C. taliensis* is also an ideal material to produce black tea with mild bitter and astringent flavor and rich polyphenols with significant antioxidant and *α*-glucosidase inhibitory activities, which indicated that the black tea from *C. taliensis* has the potential to be developed as health products.

## Figures and Tables

**Table 1:** LC-MS/MS identified compounds in black tea from *C. taliensis*.

Peak	*t*_R_ (min)	MW	MS^−^	MS^2−^	MS^+^	Compounds
1	4.04	174	173[M − H]^−^	127, 87, 45		Theanine
2	5.43	332	331[M − H]^−^	241, 211, 169[M − H − glu]^−^, 151		*β*-Glucogallin
3	6.82	170	169[M − H]^−^	125[M − COO]^−^		**19**
4	6.82	126	125[M − H]^−^			Pyrogallol
5	6.82	344	343[M − H]^−^		345[M + H]^+^	Theogallin
6	14.17	484	483[M − H]^−^	357, 313[M − H − gallic acid]^−^, 271, 210, 169, 125[169 − COO]^−^		**6**
7	15.31	184	183[M − H]^−^	169[M − CH_3_]^−^, 125[M − COOCH_3_]^−^		**23**
8	15.55	290	289[M − H]^−^	245[M − H − COO]^−^, 203[245 − C_2_H_2_O]^−^, 137[1,3A]^−^, 125[^1,4^A]^−^, 85		(+) Catechin
9	15.70	634	633[M − H]^−^	577, 463[M − H − gallic acid]^−^, 425, 387, 301[463 − glu]^−^, 274, 232, 169[gallic acid − H]^−^		**4**
10	16.12	354	353[M − H]^−^	191[quinic acid − H]^−^, 179[caffeic acid − H]^−^, 173[191 − H_2_O]^−^, 135, 69		**25**
11	18.71	290	289[M − H]^−^	245[M − H − COO]^−^, 205[M − H – 2 · C_2_H_2_O]^−^, 179[M − H − C_6_H_6_O_2_]^−^, 151[179 − CO]^−^, 125[1,4A]^−^		(−)-Epicatechin
12	19.22	458	457[M − H]^−^	411, 331, 305[M − H − C_7_H_4_O_4_]^−^, 287[M − H − C_7_H_6_O_5_]^−^, 233, 199, 169[gallic acid − H]^−^, 125[287(^1,4^A)]^−^		(−)-Epigallocatechin-3-*O*-gallate
13	19.34	338	337[M − H]^−^	191[quinic acid − H]^−^, 173[191 − H_2_O]^−^, 163[coumaric acid − H]^−^, 155, 137, 119, 93	361[M + Na]^+^	**26**
14	20.36	636	635[M − H]^−^	483[M − galloyl]^−^, 465[M − H − gallic acid]^−^, 411, 313[465 − H − galloyl]^−^, 169[gallic acid − H]^−^, 125[169 − COO]^−^		**8**
15	21.24	178	177[M − H]^−^	132, 109, 89, 65		5,7-Dihydroxycoumarin
16	23.79	610	609[M − H]^−^	466, 308[glu + rha]^−^, 301[quercetin]^−^, 300, 190, 113		**13**
17	24.37	302	301[M − H]^−^	185, 79		Not identified
18	24.85	464	463[M − H]^−^	301[quercetin]^−^, 271, 191, 169		**11**
19	24.96	442	441[M − H]^−^	331[M − H − C_6_H_6_O_2_]^−^, 289[M − galloyl]^−^, 271[M − H − gallic acid]^−^, 193[331 − C_7_H_6_O_3_]^−^, 169[gallic acid − H]^−^, 125[169 − COO]^−^	443[M + H]^+^	(−)-Epicatechin-3-*O*-gallate
20	25.25	464	463[M − H]^−^		465[M + H]^+^	Quercetin 3-*O*-*β*-D-galactopyranoside
21	25.63	594	593[M − H]^−^	447[M − rha]^−^, 285[447 − glu]^−^		**12**
22	26.31	610	609[M − H]^−^	302[quercetin]^−^		**17**
23	26.62	594	593[M − H]^−^	561, 494, 453, 413, 365, 321, 285, 230, 159, 125, 93	595[M + H]^+^	**18**
24	27.16	448	447[M − H]^−^	327, 285[M − H − glu]^−^, 255, 227, 174, 151, 127	449[M + H]^+^	**9**
25	28.21	448	447[M − H]^−^	327, 285[M − H − gal]^−^, 255, 227, 198, 151, 93	449[M + H]^+^	**14**
26	29.04	426	425[M − H]^−^	273[M − galloyl]^−^, 255[M − H − gallic acid]^−^, 229[273 − COO]^−^, 169[gallic acid − H]^−^, 151, 125[255(^1,4^A)]^−^		(−)-Epiafzelechin 3-*O*-gallate
27	31.45	432	431[M − H]^−^	285[M − H − rha]^−^, 255, 227		**15**
28	33.19	902	901[M − H]^−^	755[M − H − rha]^−^, 603, 447[755 − H − glu-cou]^−^, 416, 327, 301[447 − rha] ^−^	903[M + H]^+^	Quercetin-3-*O*-cou-rha-glu-rhamnopyranoside
29	33.90	580	579[M − H]^−^			Not identified
30	34.28	886	885[M − H]^−^	739[M − H − rha]^−^, 609, 577[739 − glu]^−^, 521, 431[577 − H − cou]^−^, 408, 285[431 − rha]^−^		Kaempferol-3-*O*-cou-rha-glu-rhamnopyranoside
31	35.95	594	593[M − H]^−^	447[M − 2H − cou]^−^, 285[447 − glu]^−^, 148		**10**
32	36.15	716	715[M − H]^−^	563[M − galloyl]^−^, 545[M − H − gallic acid]^−^, 502, 407, 319		Theaflavin-3-gallate
33	36.94	868	867[M − H]^−^	715[M − galloyl]^−^, 697[M − H − gallic acid]^−^, 571, 545[715 − gallic acid]^−^, 483, 441, 372, 257, 169		Theaflavin-3,3′-digallate
34	37.07	716	715[M − H]^−^	679, 601, 563[M − galloyl]^−^, 545[M − H − gallic acid]^−^, 316		Theaflavin-3′-digallate
35	37.24	302	301[M − H]^−^	257, 179, 151, 107		**16**

Note: cou, rha, glu, and gal refer to coumaroyl, rhamnosyl, glucosyl, and galactosyl, respectively.

**Table 2:** ^13^C (125 MHz) and ^1^H (500 MHz) NMR spectroscopic data of **1** and **4** in CD_3_OD (*δ* in ppm, *J* in Hz).

No.	1		4	
	*δ*_C_, Type	*δ*_H_ (*J* in Hz)	*δ*_C_, Type	*δ*_H_ (*J* in Hz)
Glucose-1	95.7, d	5.73, d, *J* = 8.2 Hz	96.2, s	5.69, d, *J* = 8.1 Hz
2	74.2, d	3.65, dd, *J* = 9.4, 8.2 Hz	74.7, t	3.63, dd, *J* = 9.4, 8.1 Hz
3	74.9, d	3.86, t, *J* = 9.4 Hz	76.0, d	3.74, t, *J* = 9.4 Hz
4	76.2, d	5.00, m	73.2, t	4.87, t, *J* = 9.4 Hz
5	71.7, d	3.98, m	73.7, d	4.06, dd, *J* = 9.4, 6.5 Hz
6	67.6, t	a 4.78, m	64.3, t	a 5.24, dd, *J* = 13.3, 6.5 Hz
		b 4.01, m		b 3.83, dd, *J* = 13.3, 1.2 Hz
Galloyl-1′	120.4, s		120.5, s	
2′,6′	110.6, d	7.12, s	110.5, d	7.16, s
3′,5′	146.6, s		146.6, s	
4′	140.6, s		140.5, s	
7′	166.8, s		166.8, s	
Acyl-1″	115.9, s		116.8, s	
2″	117.0, s		126.3, s	
3″	116.2, s	7.20, s	108.6, d	6.71, s
4″	145.2, s		145.9, s	
5″	136.1, s		137.6, s	
6″	147.6, s		144.9, s	
7″	170.0, s		169.6, s	
Acyl-1‴	115.4, s		116.6, s	
2‴	119.8, s		126.6, s	
3‴	112.1, s	7.05, s	108.3, d	6.57, s
4‴	145.5, s		145.8, s	
5‴	134.0, s		137.3, s	
6‴	147.7, s		144.8, s	
7‴	171.5, s		169.9, s	

**Table 3:** Antioxidant activities of compounds **1**–**8**, **9**–**18**, **19**–**23,** and **25**–**32** from black tea from *C. taliensis* ^a^.

Samples	DPPH		ABTS^+^	
	IC_50_ (μM) ^b^	Inhibition Ratio (%) ^c^	IC_50_ (μM) ^b^	Inhibition Ratio (%) ^c^
**1**	338.9 ± 24.7		110.7 ± 2.1	
**2**	502.1 ± 14.0		400.4 ± 9.6	
**3**	941.3 ± 191.0		106.0 ± 0.5	
**4**	311.5 ± 10.8		84.8 ± 0.4	
**5**	300.8 ± 79.6		75.6 ± 2.1	
**6**	217.3 ± 27.4		99.7 ± 1.4	
**7**	532.1 ± 38.0		128.4 ± 0.9	
**8**	156.5 ± 26.1		77.2 ± 0.7	
**9**		0.97		0.19
**10**		6.28		11.55
**11**	2304.0 ± 372.3		322.8 ± 0.6	
**12**		8.58		4.24
**13**	900.3 ± 63.4		265.8 ± 5.4	
**14**		−0.94		4.03
**15**		1.04		8.00
**16**	1141.6 ± 206.8		206.2 ± 2.6	
**17**	2616.0 ± 500.5		480.9 ± 4.1	
**18**		1.08		5.89
**19**	237.2 ± 22.6		201.7 ± 3.4	
**20**	2119.3 ± 335.7		462.0 ± 9.3	
**21**		3.32		3.21
**22**		7.58		7.00
**23**	298.8 ± 32.3		187.4 ± 2.7	
**25**	455.5 ± 45.5		272.0 ± 1.6	
**26**		6.92		6.82
**27**	722.2 ± 43.2		545.1 ± 12.8	
**28**	478.7 ± 33.8		198.7 ± 4.1	
**29**		1.55		8.12
**30**	547.5 ± 61.5		306.9 ± 3.3	
**31**	335.2 ± 24.0		107.4 ± 0.5	
**32**	3938.3 ± 825.2		560.3 ± 3.3	
Ascorbic acid ^d^	1197.0 ± 87.7			
Trolox ^d^			404.8 ± 6.2	

^a^ Values represent the means ± SD (n = 3). ^b^ IC_50_ = half-maximal inhibitory concentration in μM to DPPH and ABTS^+^ radical. ^c^ Inhibition ratio (%) at a concentration of 500 μM. ^d^ Positive control.

**Table 4:** The inhibitory activities of **1**–**8** and **19** on *α*-glucosidase in black tea from *C. taliensis* ^a^.

Samples	IC_50_ (μM) ^b^	Inhibition Ratio (%) ^c^
Quercetin ^d^	5.75 ± 0.78	64.81 ± 3.30 ^e^
Acarbose ^d^	223.30 ± 9.98	65.02 ± 1.19 ^f^
**1**	1.77 ± 0.05	
**2**		18.29 ± 1.33
**3**	1.74 ± 0.03	
**4**	1.96 ± 0.06	
**5**	0.67 ± 0.04	
**6**		38.42 ± 1.57
**7**	1.96 ± 0.05	
**8**	2.01 ± 0.02	
**19**		3.01 ± 1.66

^a^ Values represent means ± SD (n = 3). ^b^ IC_50_ = half-maximal inhibitory concentration to *α*-glucosidase. ^c^ Inhibition ratio (%) at a concentration of 50 μM. ^d^ Positive control. ^e^ Tested concentration: 10 μM. ^f^ Tested concentration: 400 μM.

## Data Availability

Data are contained within the article or supplementary material.

## Supplementary Materials

The following supporting information can be downloaded at: https://www.mdpi.com/article/10.3390/foods12132512/s1, Figure S1: ^1^H NMR spectrum of compound **1** in CD_3_OD; Figure S2: ^13^C NMR spectrum of compound **1** in CD_3_OD; Figure S3: HSQC spectrum of compound **1** in CD_3_OD; Figure S4: HMBC spectrum of compound **1** in CD_3_OD; Figure S5: COSY spectrum of compound **1** in CD_3_OD; Figure S6: ROESY spectrum of compound **1** in CD_3_OD; Figure S7: HRESI-MS spectrum of compound **1**; Figure S8: CD and UV spectra of compound **1** in MeOH; Figure S9: OR of compound **1** in MeOH; Figure S10: Previous studies on the oxidative coupling of galloyl groups; Figure S11: ^1^H NMR spectrum of compound **2** in CD_3_OD; Figure S12: ^13^C NMR spectrum of compound **2** in CD_3_OD; Figure S13: ^1^H NMR spectrum of compound **3** in CD_3_OD; Figure S14: ^13^C NMR spectrum of compound **3** in CD_3_OD; Figure S15: ^1^H NMR spectrum of compound **4** in CD_3_OD; Figure S16: ^13^C NMR spectrum of compound **4** in CD_3_OD; Figure S17: ^1^H NMR spectrum of compound **5** in CD_3_OD; Figure S18: ^13^C NMR spectrum of compound **5** in CD_3_OD; Figure S19: ^1^H NMR spectrum of compound **6** in CD_3_OD; Figure S20: ^13^C NMR spectrum of compound **6** in CD_3_OD; Figure S21: ^1^H NMR spectrum of compound **7** in CD_3_OD; Figure S22: ^13^C NMR spectrum of compound **7** in CD_3_OD; Figure S23: ^1^H NMR spectrum of compound **8** in CD_3_OD; Figure S24: ^13^C NMR spectrum of compound **8** in CD_3_OD.
