# Supplementary material for: New Hydrolyzable Tannin with Potent Antioxidant and α-Glucosidase Inhibitory Activity from Black Tea Produced from Camellia taliensis
Source: Foods. 2023 Jun 28;12(13):2512. doi: 10.3390/foods12132512 (PMC10340768; doi:10.3390/foods12132512)

Supplementary data contents page

New hydrolyzable tannin with potent antioxidant and *α*-glucosidase inhibitory activity from black tea produced from *Camellia taliensis*

Min Chen[1,2,†], Na Li[1,†], Hong-Tao Zhu[1], Man Zhang[1], Zhao-Hong Duan[3], Dong Wang[1], Chong-Ren Yang[1], and Ying-Jun Zhang[1,*]

[1] *State Key Laboratory of Phytochemistry and Plant Resources in West China, Kunming Institute of Botany, Chinese Academy of Sciences, Kunming 650201, People's Republic of China*

[2] *University of Chinese Academy of Sciences, Beijing 100049, People's Republic of China*

[3] *Zhao-Hong Gu-Shu Training Center, Lincang 675911, People's Republic of China*

† *These authors contributed equally to this work*

* *Correspondence: zhangyj@mail.kib.ac.cn (Y.J.Z.); Tel.: + 86 871 6522 3235*

1. Figure S1. ^{1}H NMR spectrum of compound **1** in CD_3OD
2. Figure S2. ^{13}C NMR spectrum of compound **1** in CD_3OD
3. Figure S3. HSQC spectrum of compound **1** in CD_3OD
4. Figure S4. HMBC spectrum of compound **1** in CD_3OD
5. Figure S5. COSY spectrum of compound **1** in CD_3OD
6. Figure S6. ROESY spectrum of compound **1** in CD_3OD
7. Figure S7. HRESI-MS spectrum of compound **1**
8. Figure S8. CD and UV spectra of compound **1** in MeOH
9. Figure S9. OR of compound **1** in MeOH
10. Figure S10. Previous studies on the oxidative coupling of galloyl groups
11. Figure S11. ^{1}H NMR spectrum of compound **2** in CD_3OD
12. Figure S12. ^{13}C NMR spectrum of compound **2** in CD_3OD
13. Figure S13. ^{1}H NMR spectrum of compound **3** in CD_3OD
14. Figure S14. ^{13}C NMR spectrum of compound **3** in CD_3OD
15. Figure S15. ^{1}H NMR spectrum of compound **4** in CD_3OD
16. Figure S16. ^{13}C NMR spectrum of compound **4** in CD_3OD
17. Figure S17. ^{1}H NMR spectrum of compound **5** in CD_3OD

18. Figure S18. ^{13}C NMR spectrum of compound **5** in CD_3OD
19. Figure S19. ^{1}H NMR spectrum of compound **6** in CD_3OD
20. Figure S20. ^{13}C NMR spectrum of compound **6** in CD_3OD
21. Figure S21. ^{1}H NMR spectrum of compound **7** in CD_3OD
22. Figure S22. ^{13}C NMR spectrum of compound **7** in CD_3OD
23. Figure S23. ^{1}H NMR spectrum of compound **8** in CD_3OD
24. Figure S24. ^{13}C NMR spectrum of compound **8** in CD_3OD

Figure S1. ^{1}H NMR spectrum of compound **1** in CD_3OD

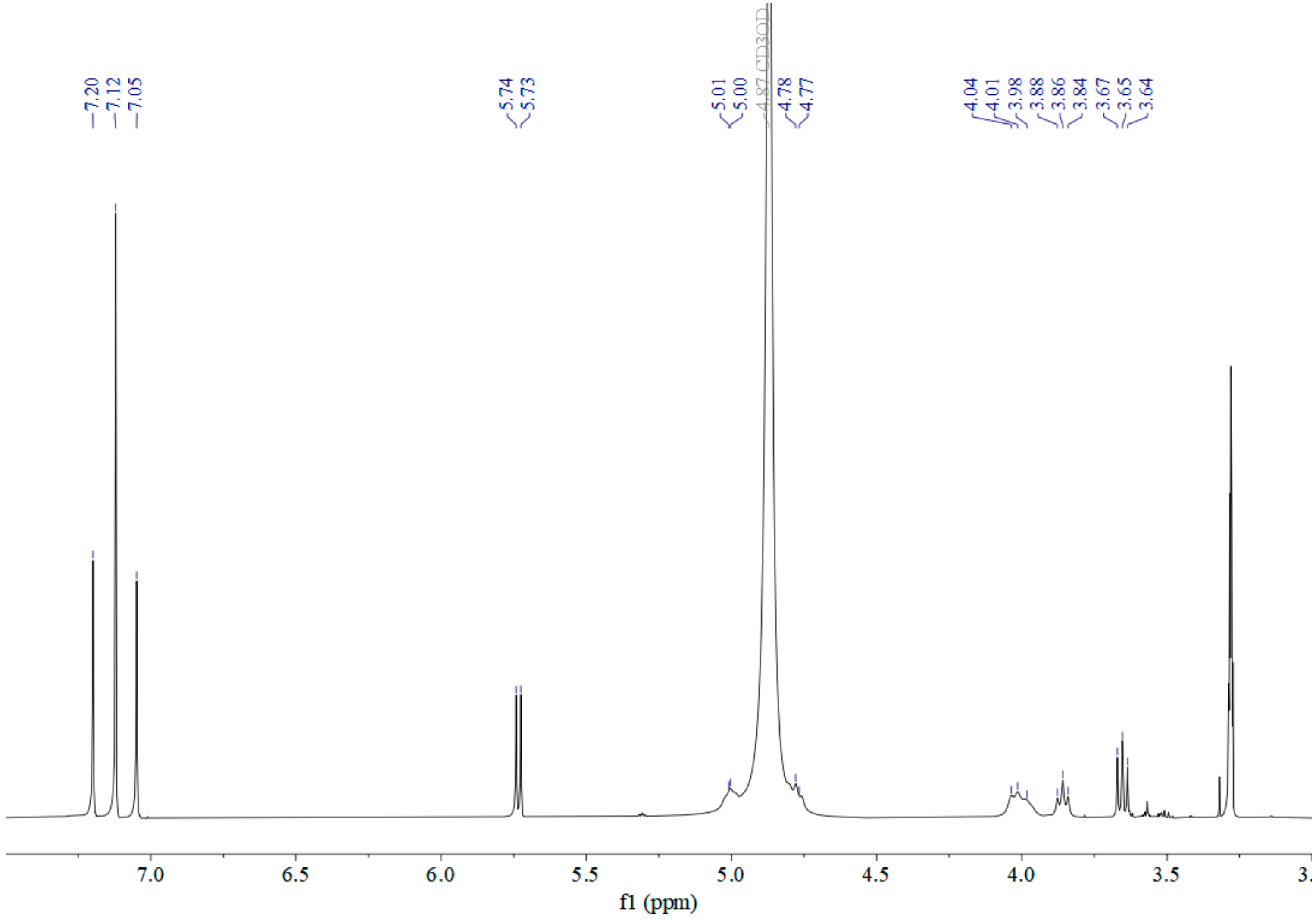

Figure S2. ^{13}C NMR spectrum of compound **1** in CD_3OD

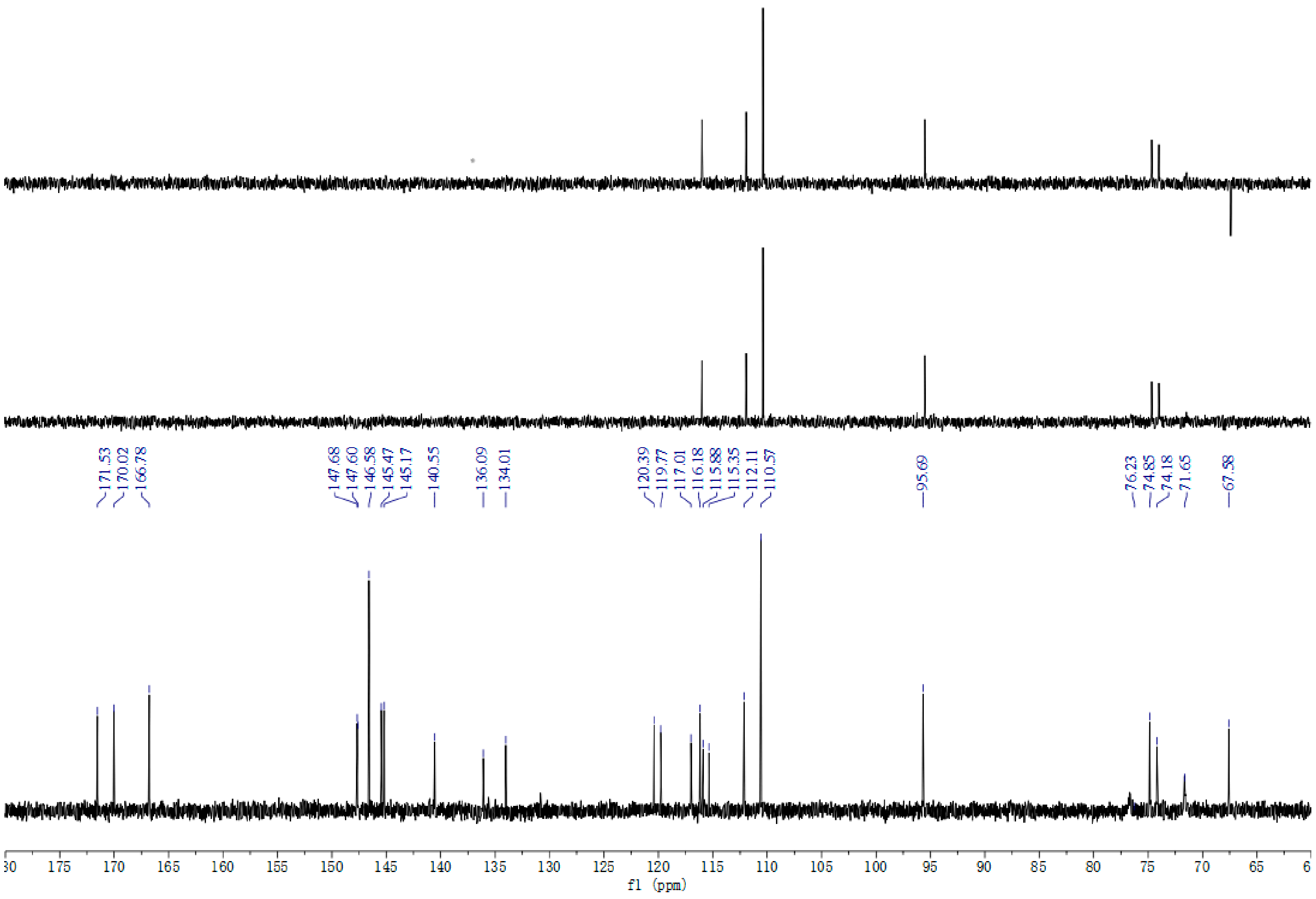

Figure S3. HSQC spectrum of compound **1** in CD_3OD

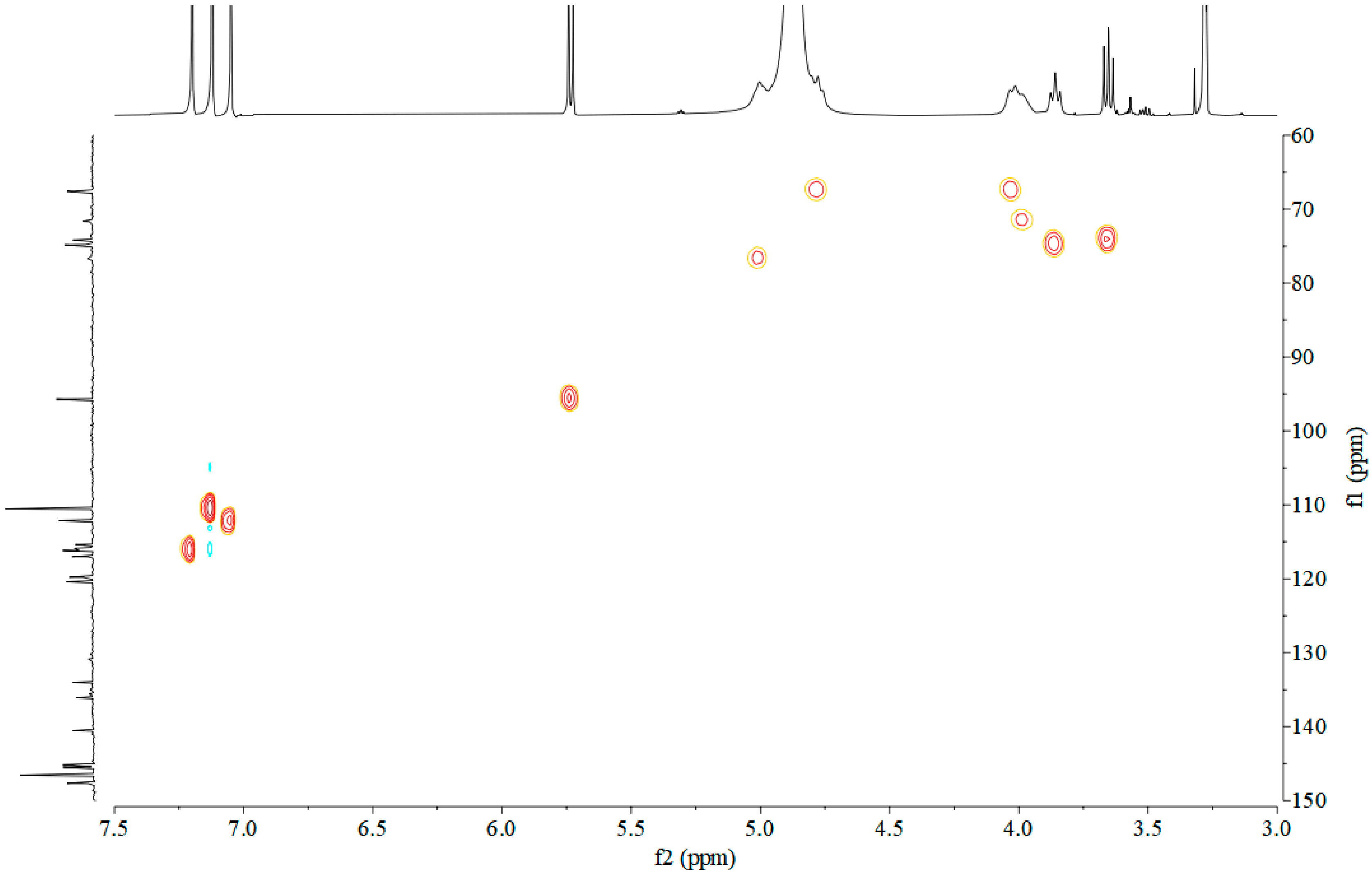

Figure S4. HMBC spectrum of compound **1** in CD_3OD

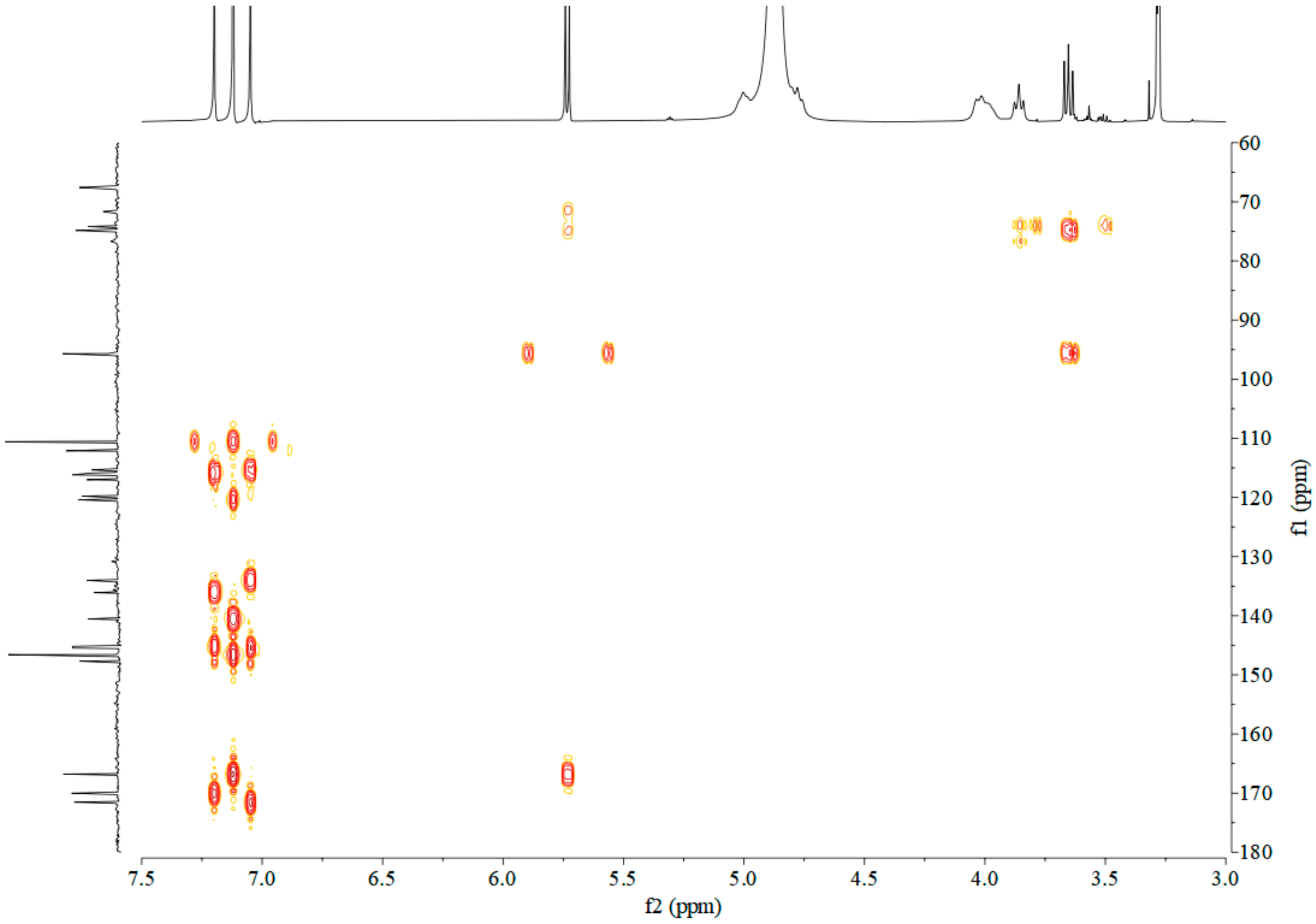

Figure S5. COSY spectrum of compound **1** in CD_3OD

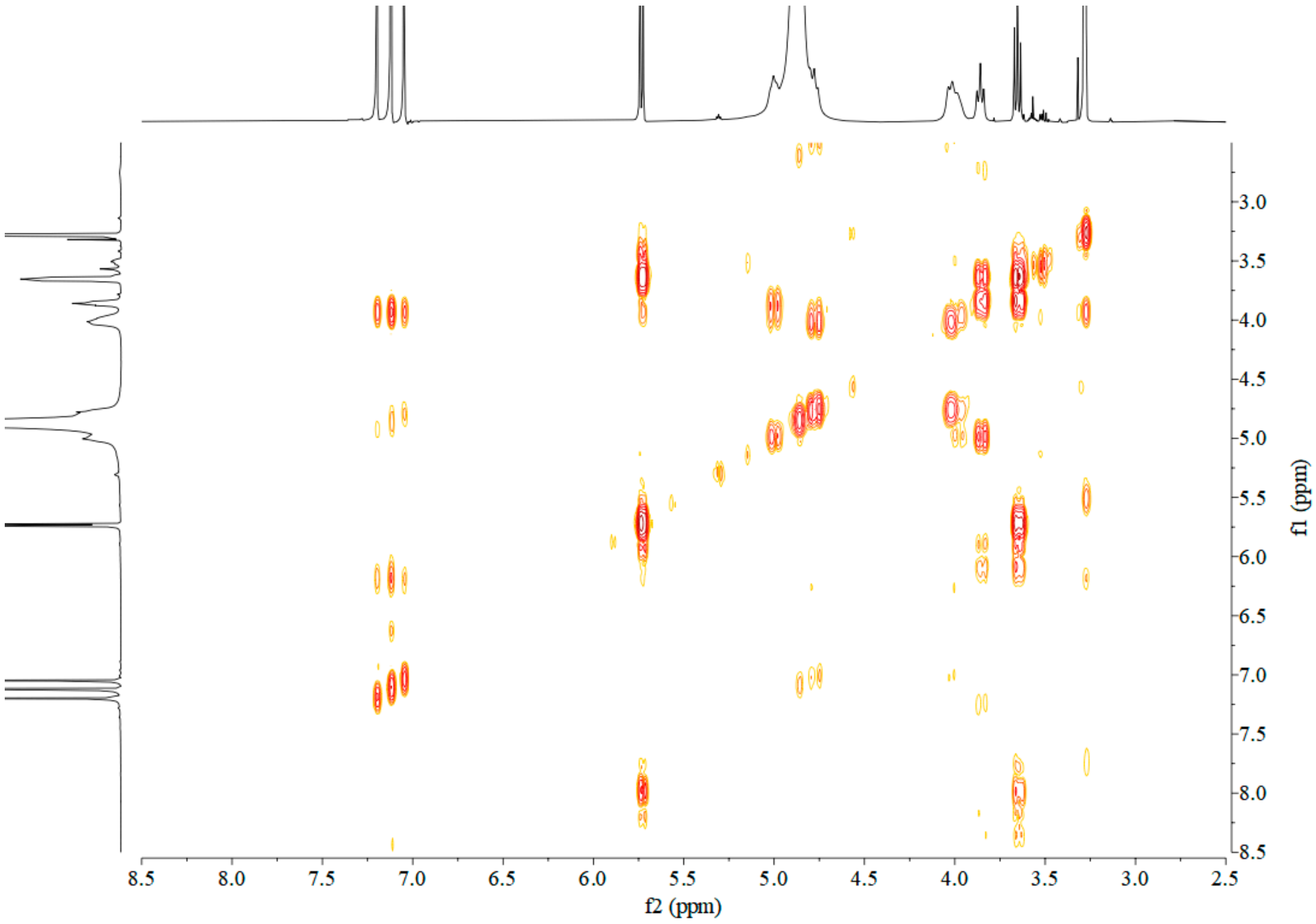

Figure S6. ROESY spectrum of compound **1** in CD_3OD

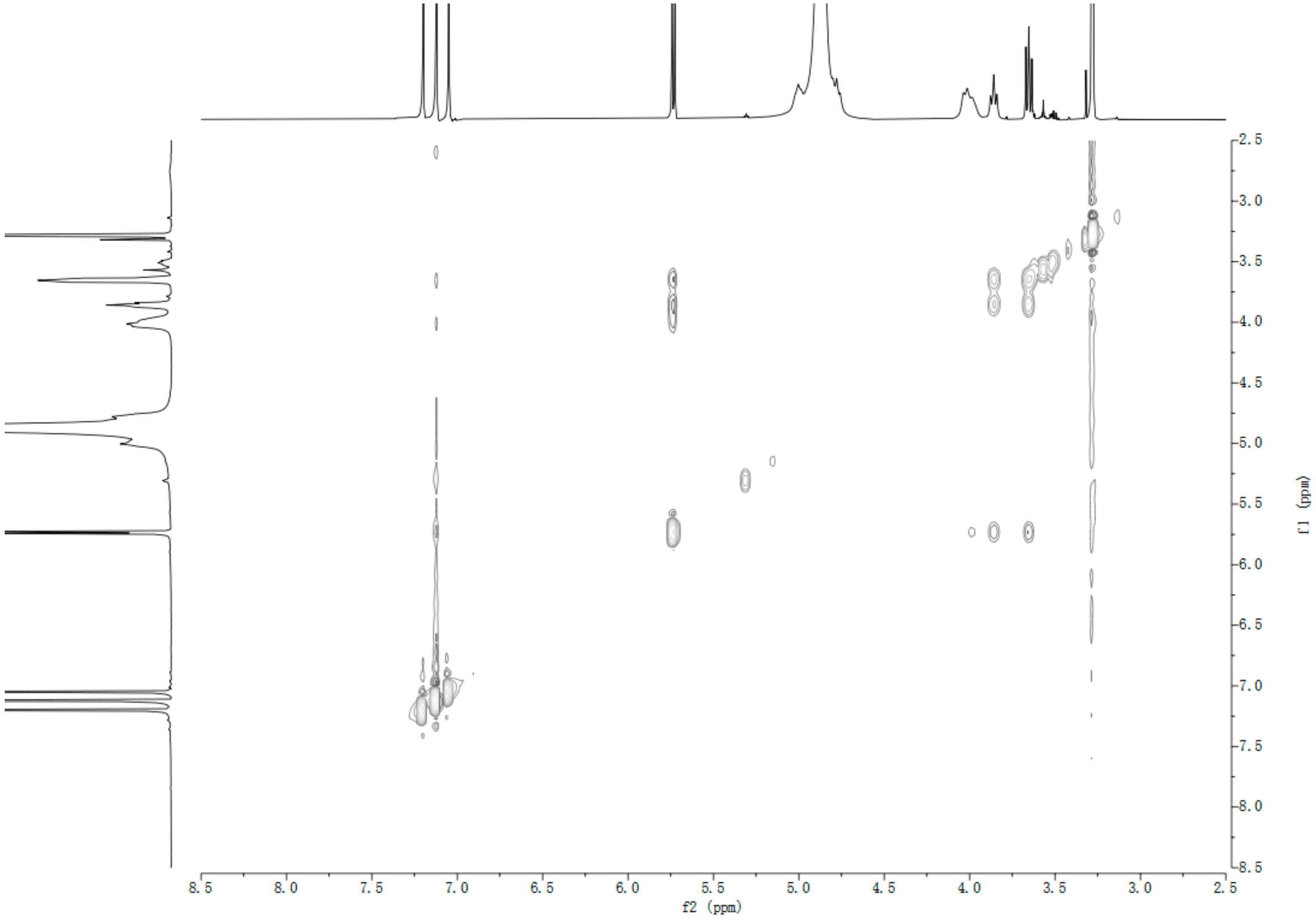

Figure S7. HRESI-MS spectrum of compound **1**

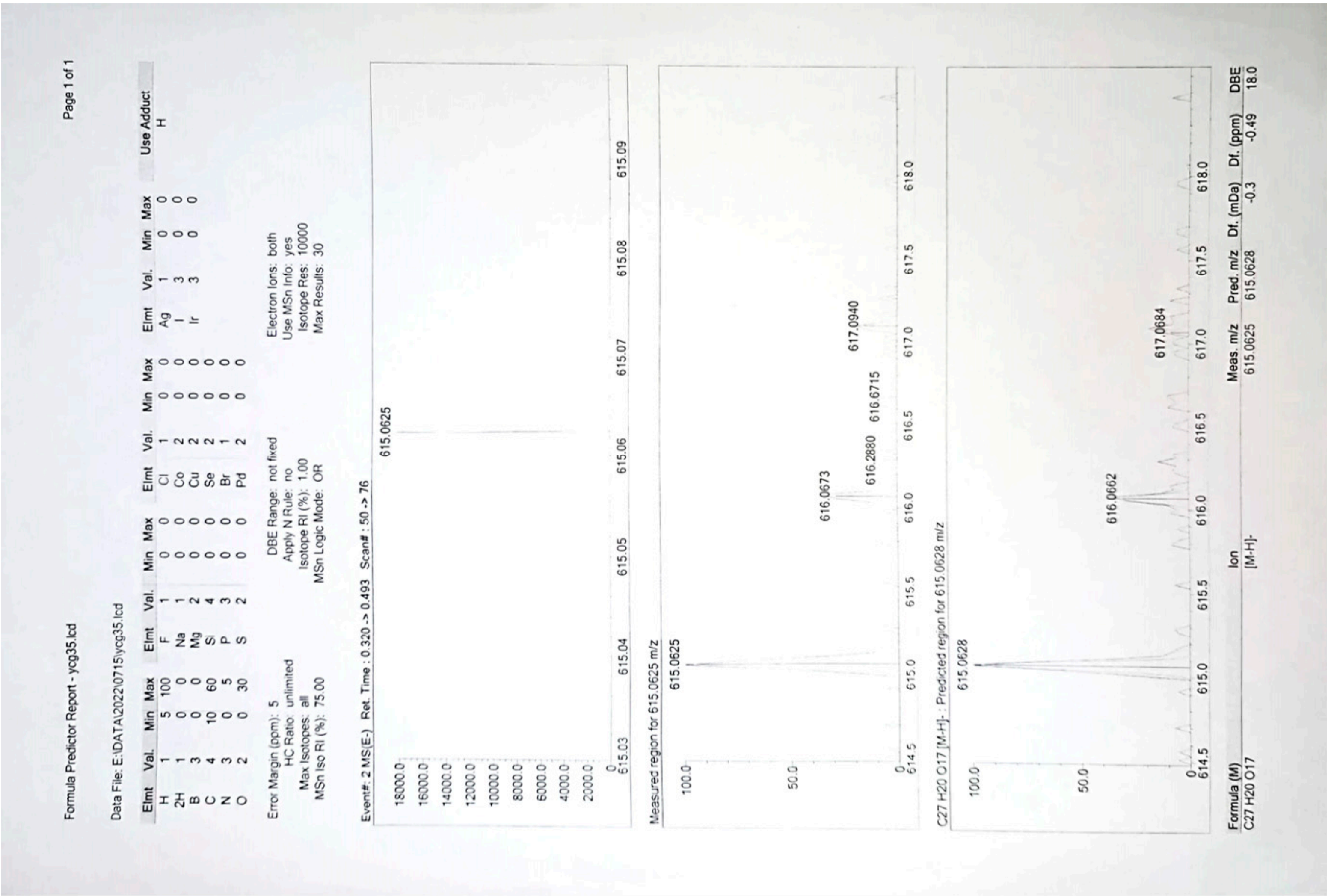

Figure S8. CD and UV spectra of compound **1** in MeOH

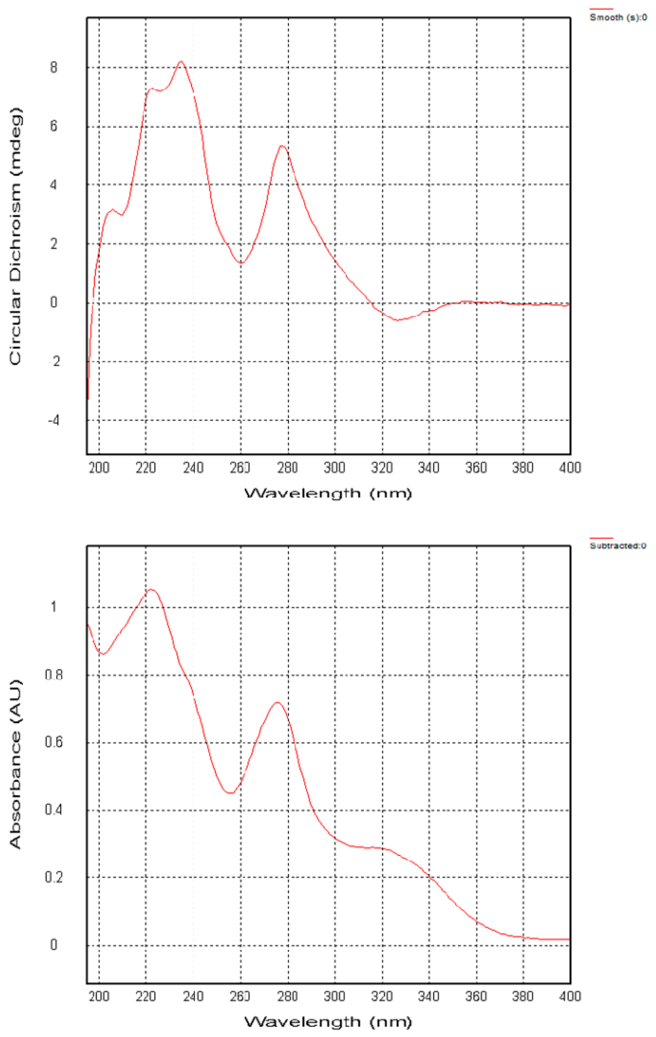

File: YCG35-1mm(195-400)22092905.dsx
ProBinaryX
Attributcs :
- Time Stamp :Thu Sep 29 14:14:13 2022

- File ID : {A6D38CEA-A099-42ac-B679-26193C9FEFD4}
- Is CFR Compliant : false
- Original unaltered data

Remarks:
- User: CD
- Date: 2022/09/29
- Instrument: 0547
- DetectorType: LAAPD
- DichOS Calibration Correction Curve: 0547/2
- HV (CDDC channel): 0 v
- Time per point: 1 s
- Description: Sample 1
- Concentration: 0.2000mg/mLMeOH
- Pathlength: 1 mm
- Temperature: 20°C

Settings:
- HV
- Time-per-point: 1s (25us x 40000)
- SE
- Wavelength: 195nm - 400nm
- Step Size: 1nm
- Bandwidth: 1nm

Figure S9. OR of compound **1** in MeOH

Rudolph Research Analytical

This sample was measured on an Autopol VI, Serial #91058
Manufactured by Rudolph Research Analytical, Hackettstown, NJ, USA.

Measurement Date : Wednesday, 28-SEP-2022

Set Temperature : OFF

Time Delay : Disabled

Delay between Measurement : Disabled

n	Average	Std.Dev.	% RSD	Maximum	Minimum
5	34.40	0.55	1.59	35.00	34.00

S.No	Sample ID	Time	Result	Scale	OR °Arc	WLG.nm	Lg.mm	Conc.g/100ml	Temp.
1	YCG35	04:48:48 PM	35.00	SR	0.035	589	100.00	0.100	23.6
2	YCG35	04:48:54 PM	35.00	SR	0.035	589	100.00	0.100	23.6
3	YCG35	04:49:01 PM	34.00	SR	0.034	589	100.00	0.100	23.6
4	YCG35	04:49:07 PM	34.00	SR	0.034	589	100.00	0.100	23.6
5	YCG35	04:49:13 PM	34.00	SR	0.034	589	100.00	0.100	23.6

Figure S10. Previous studies on the oxidative coupling of galloyl groups

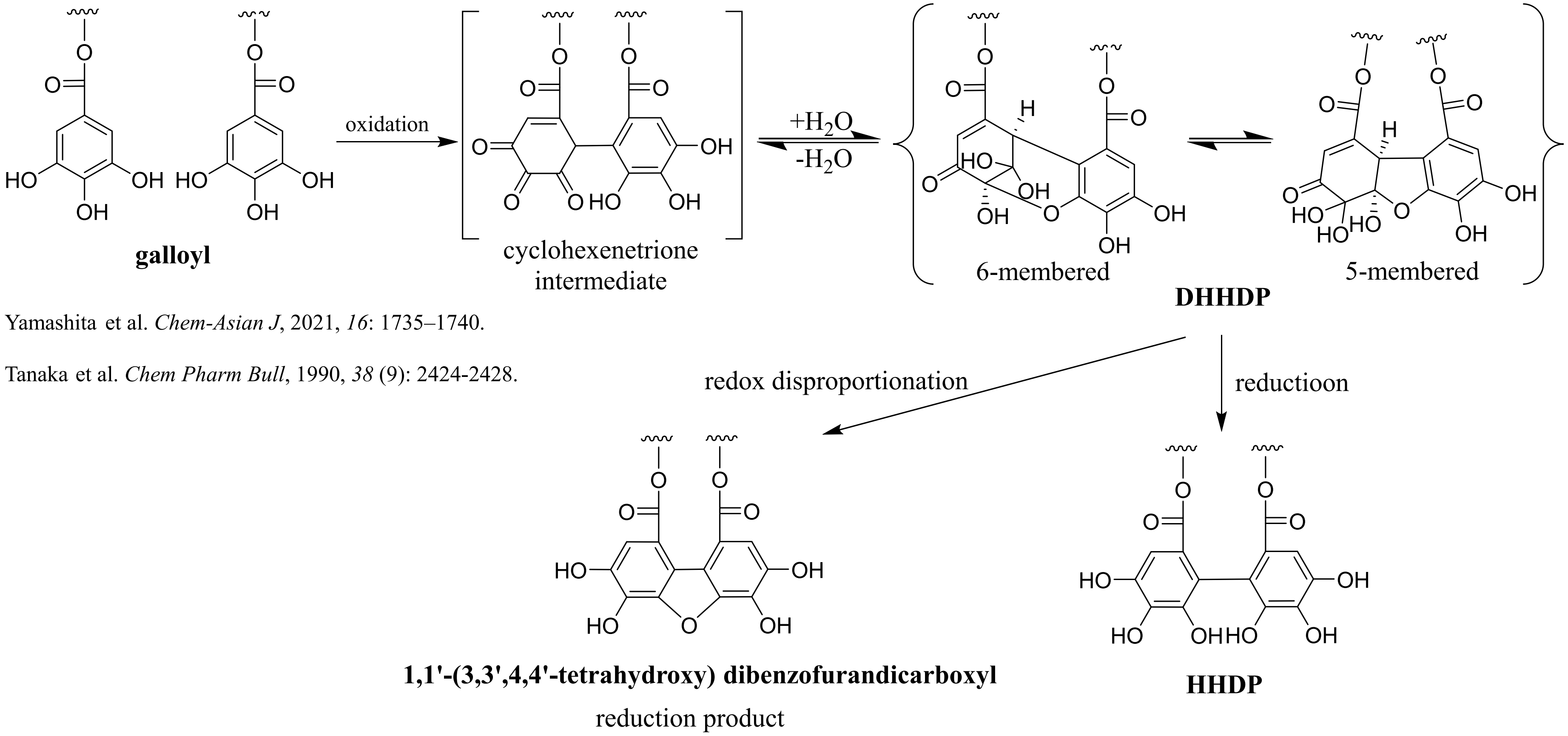

Yamashita et al. *Chem-Asian J*, 2021, *16*: 1735–1740.

Tanaka et al. *Chem Pharm Bull*, 1990, *38* (9): 2424-2428.

Figure S11. ^{1}H NMR spectrum of compound **2** in CD_3OD

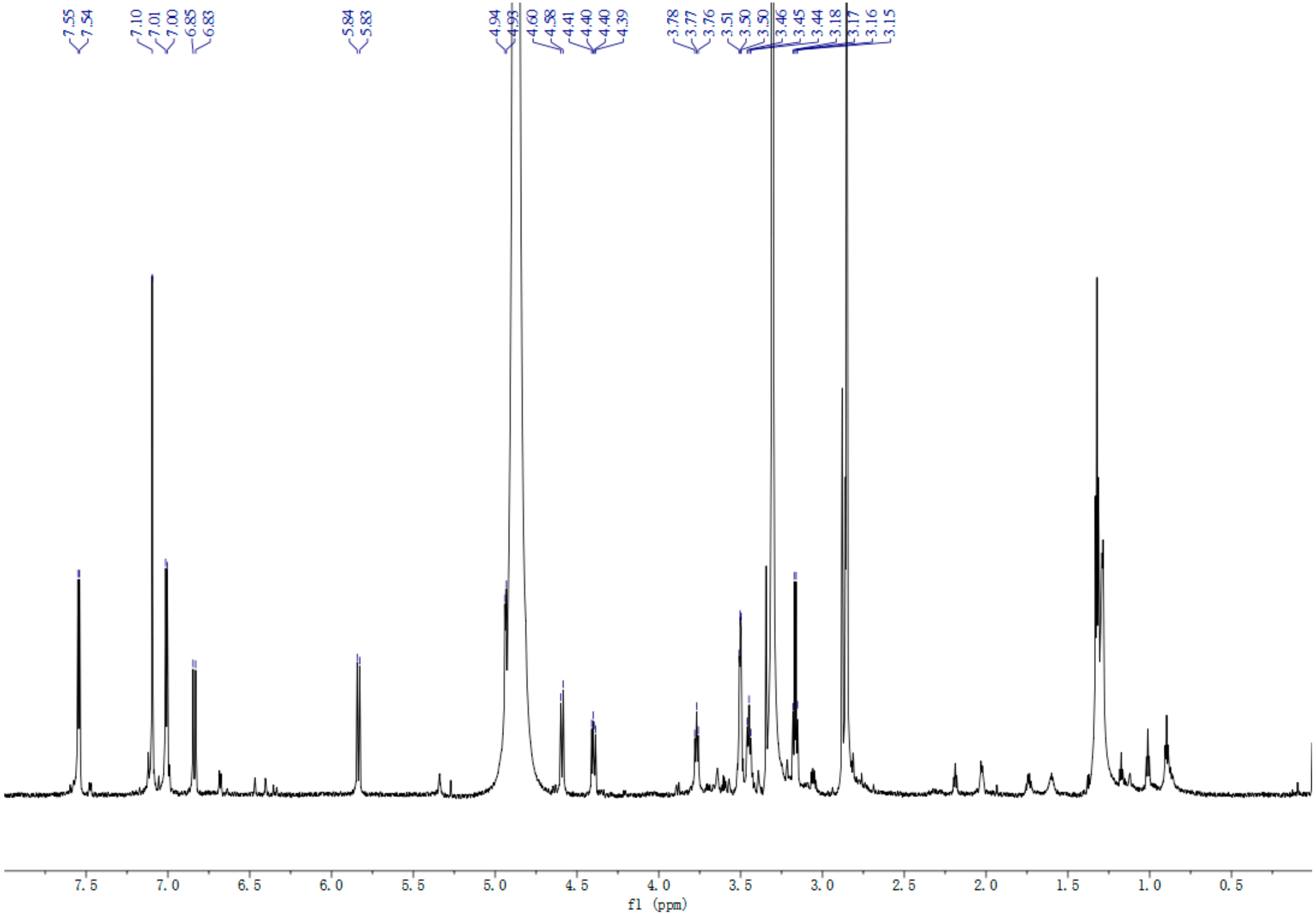

Figure S12. ^{13}C NMR spectrum of compound **2** in CD_3OD

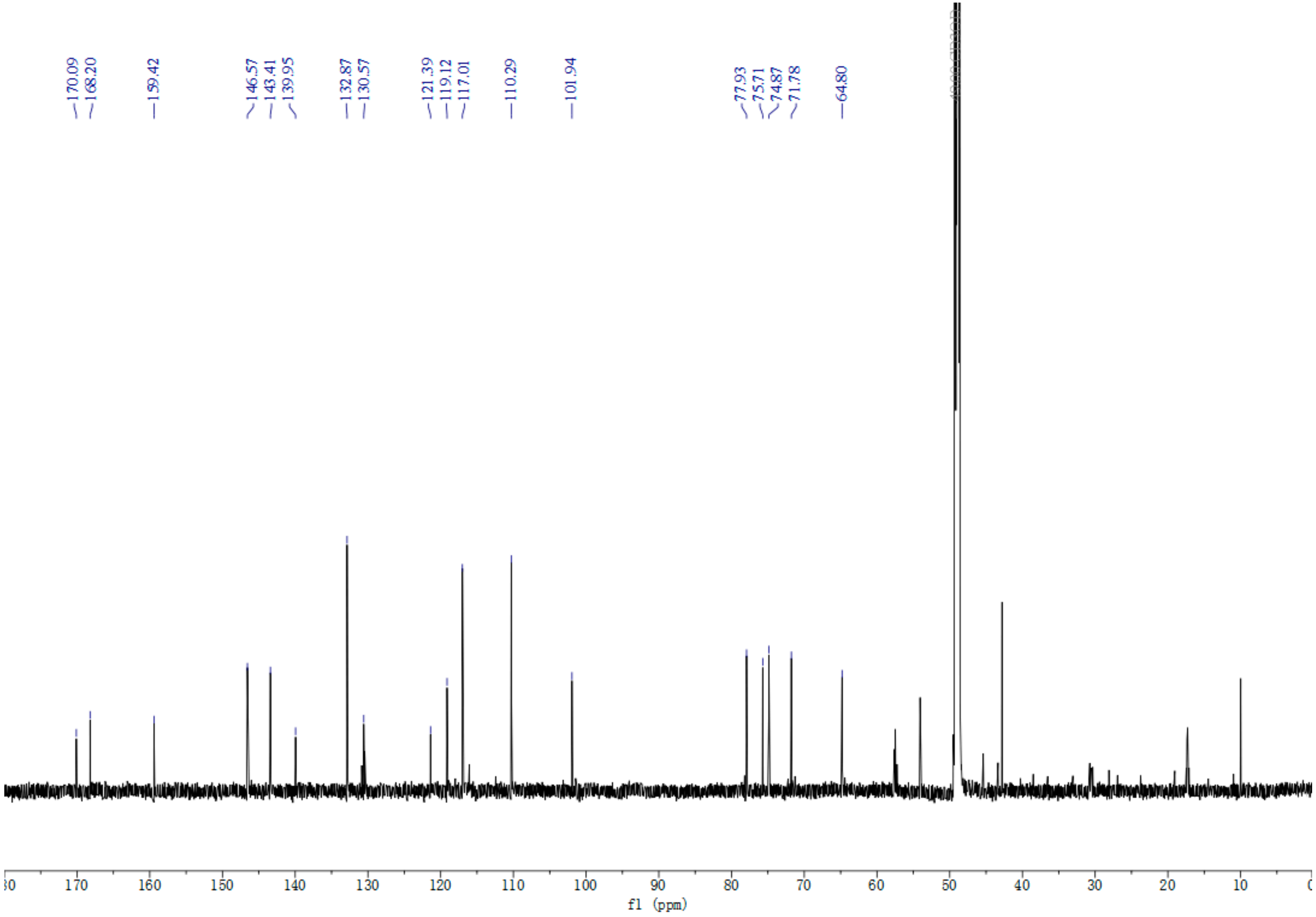

Figure S13. ^{1}H NMR spectrum of compound **3** in CD_3OD

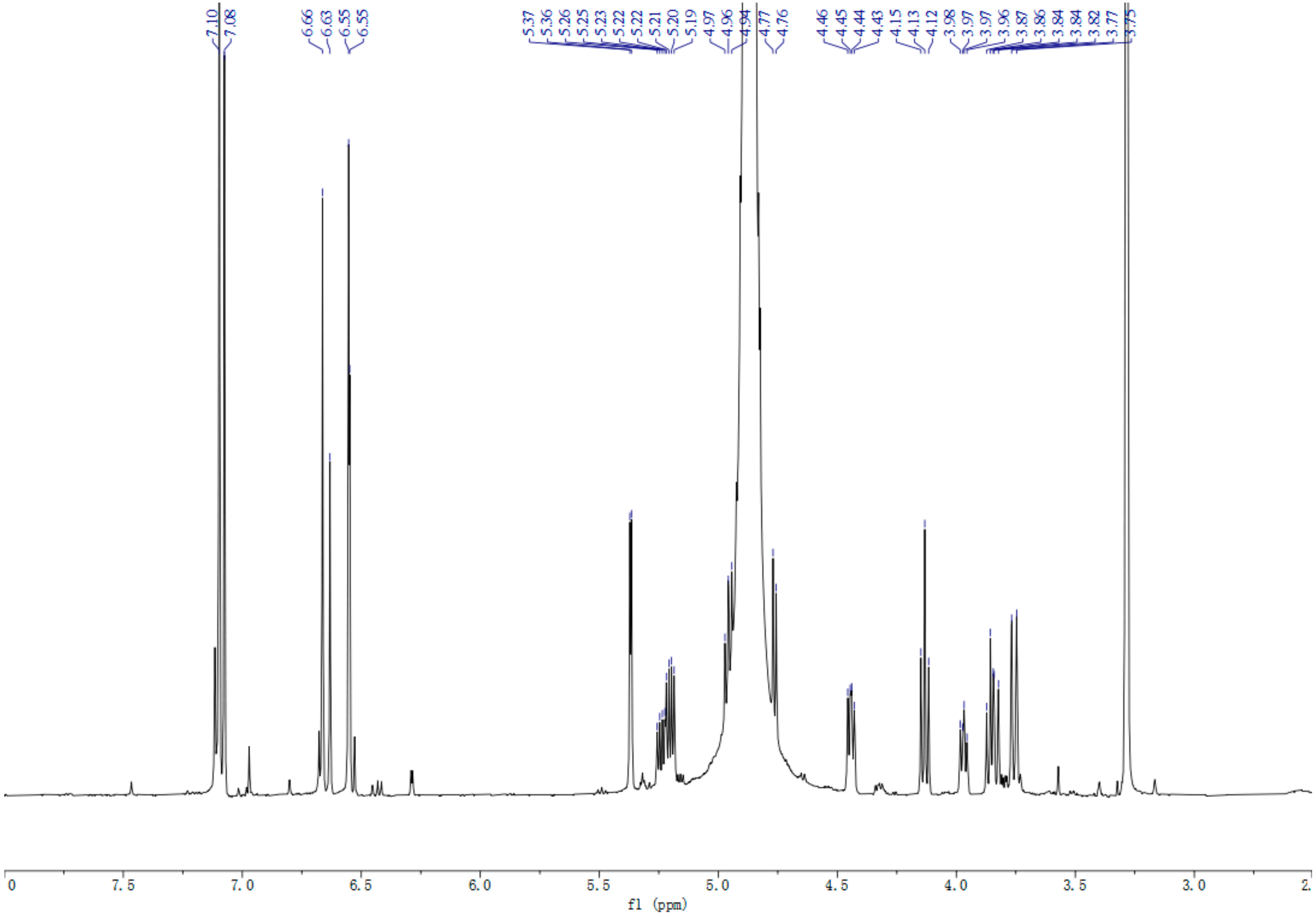

Figure S14. ^{13}C NMR spectrum of compound **3** in CD_3OD

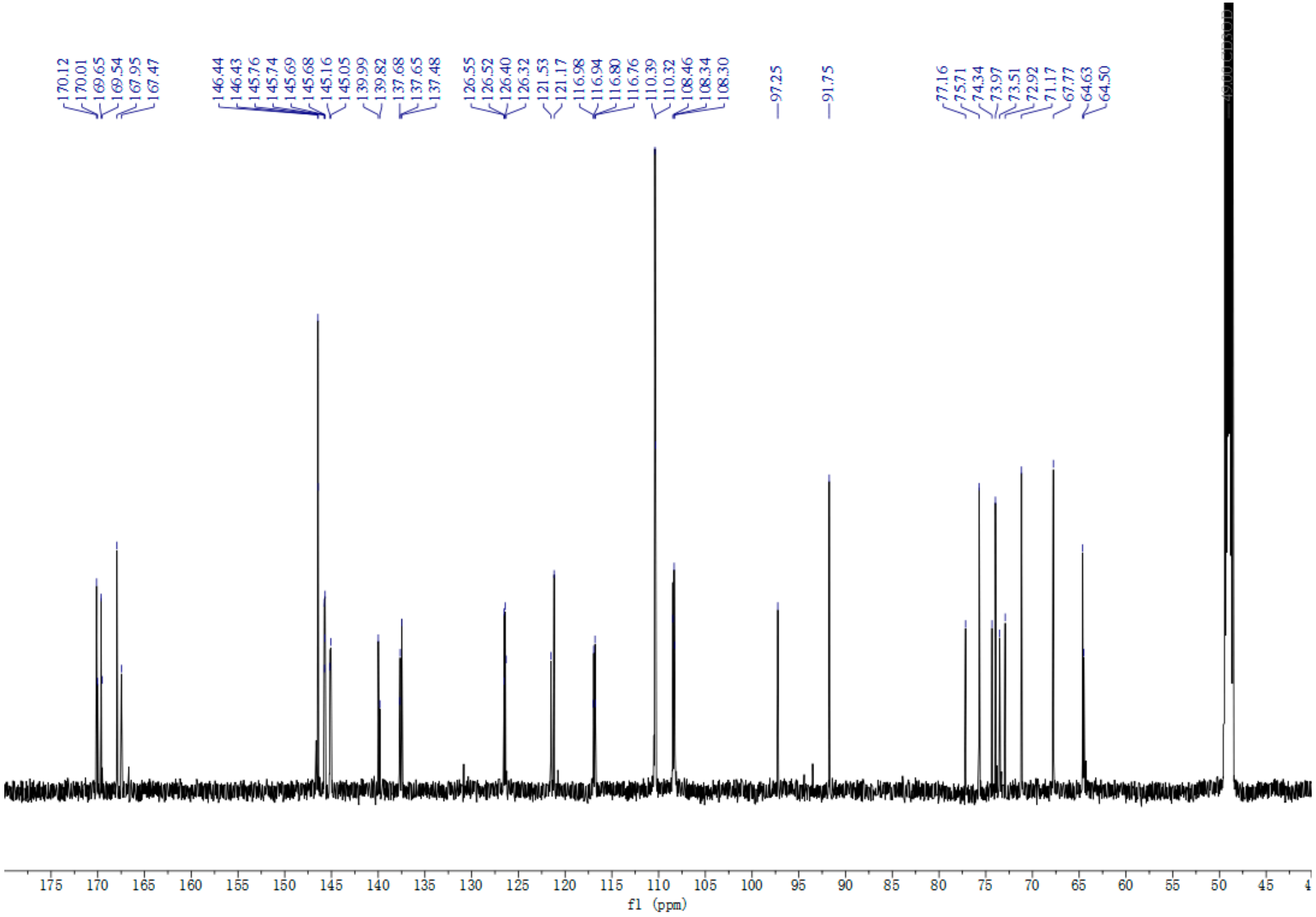

Figure S15. ^{1}H NMR spectrum of compound **4** in CD_3OD

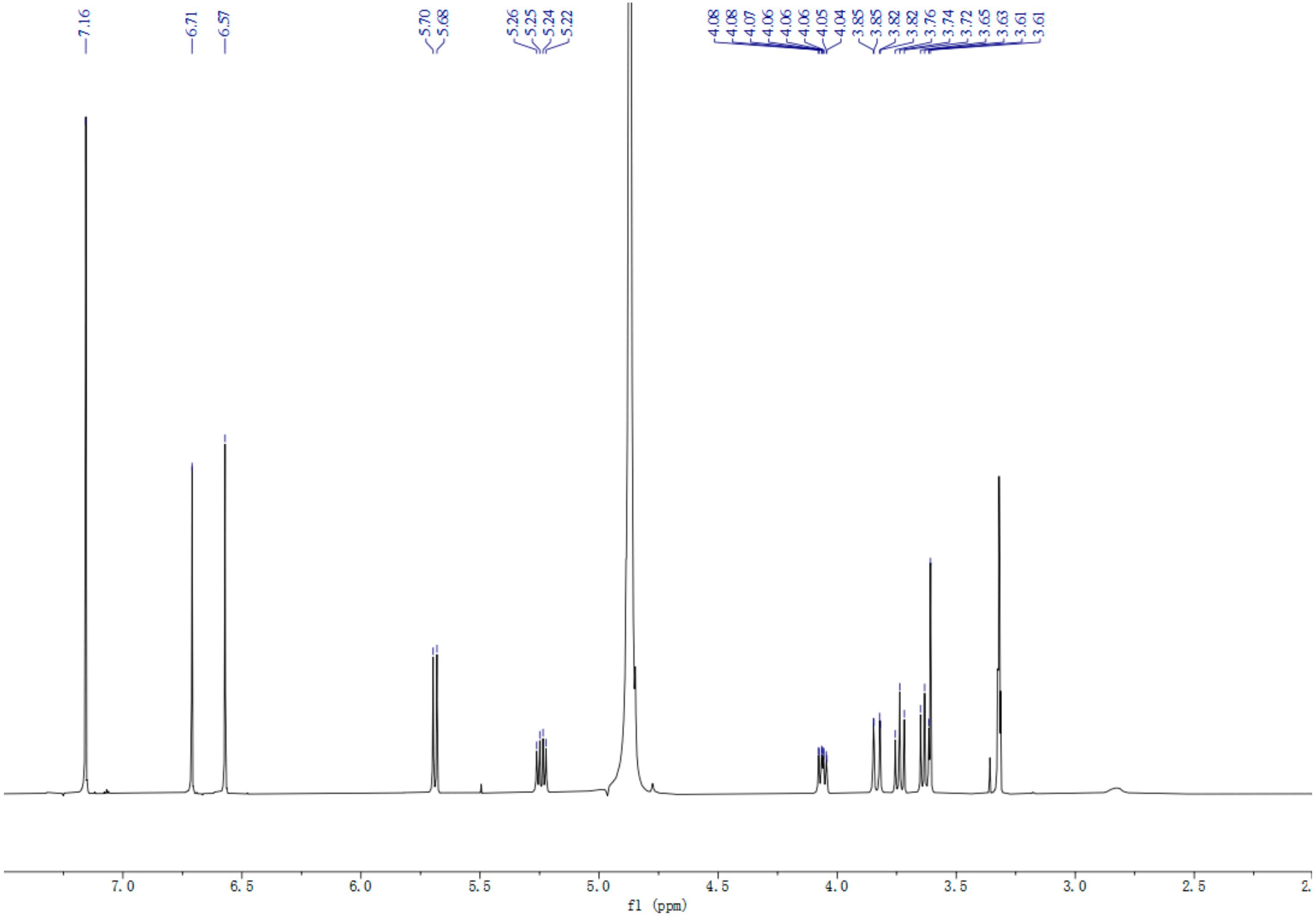

Figure S16. ^{13}C NMR spectrum of compound **4** in CD_3OD

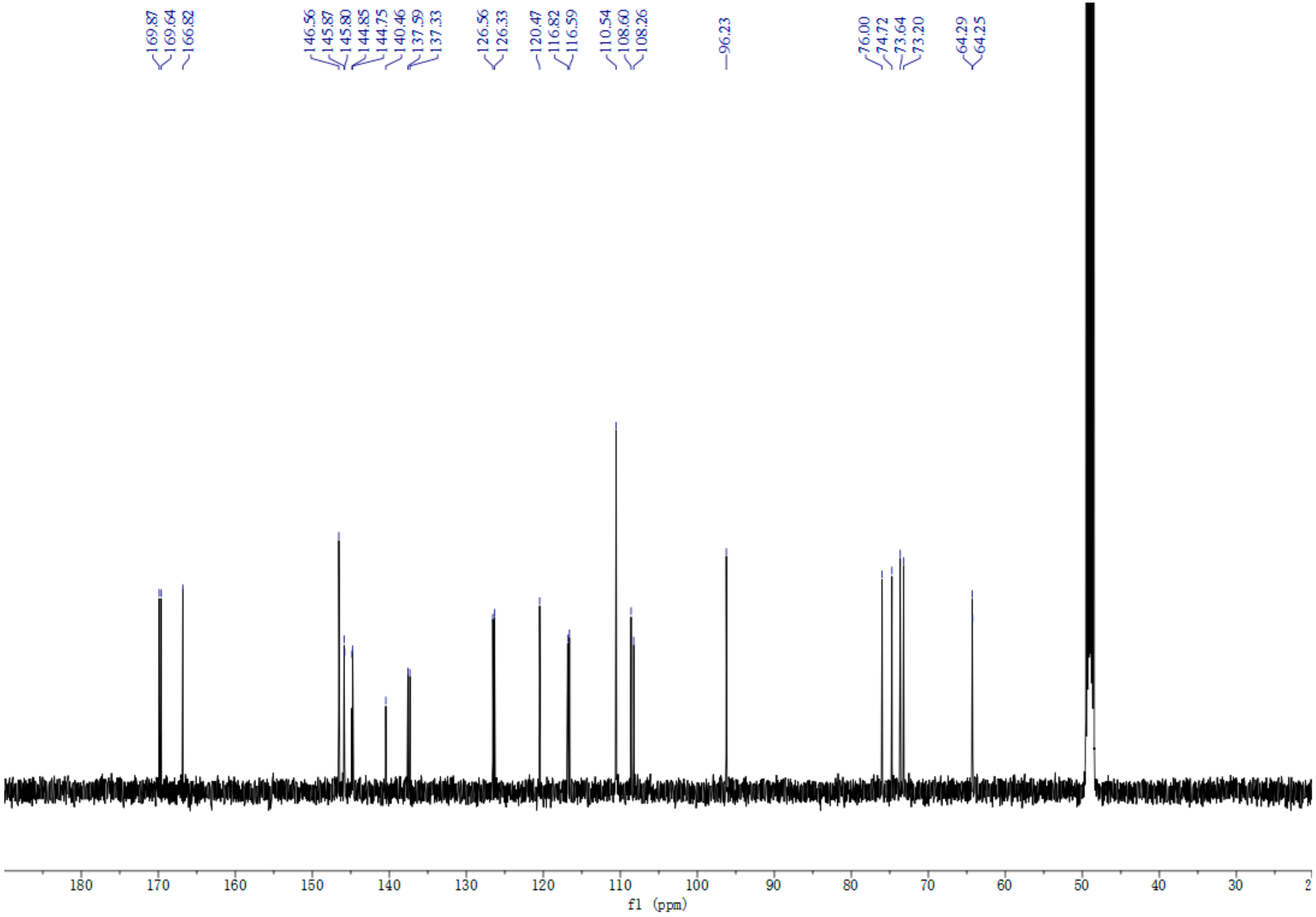

Figure S17. ^{1}H NMR spectrum of compound **5** in CD_3OD

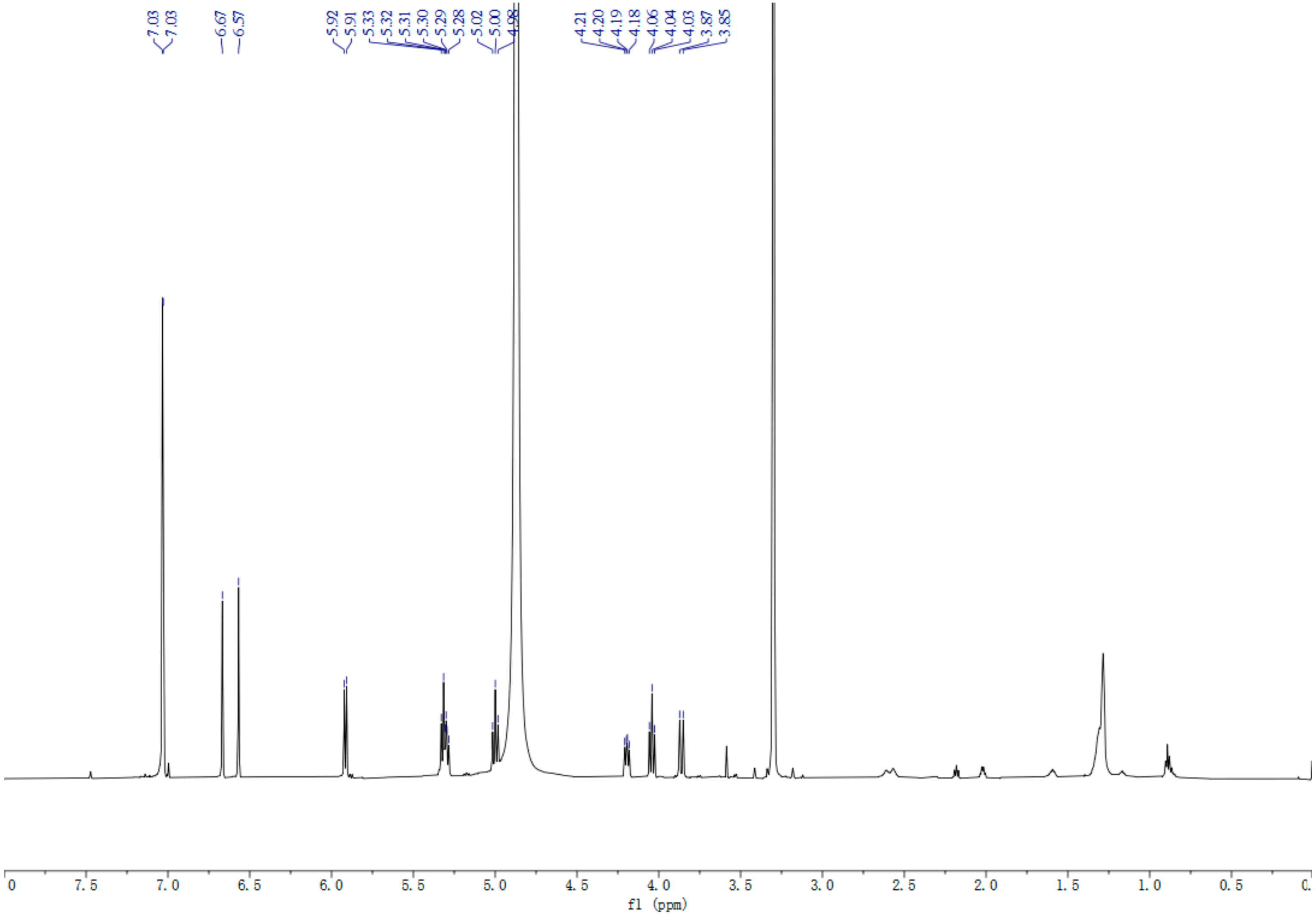

Figure S18. ^{13}C NMR spectrum of compound **5** in CD_3OD

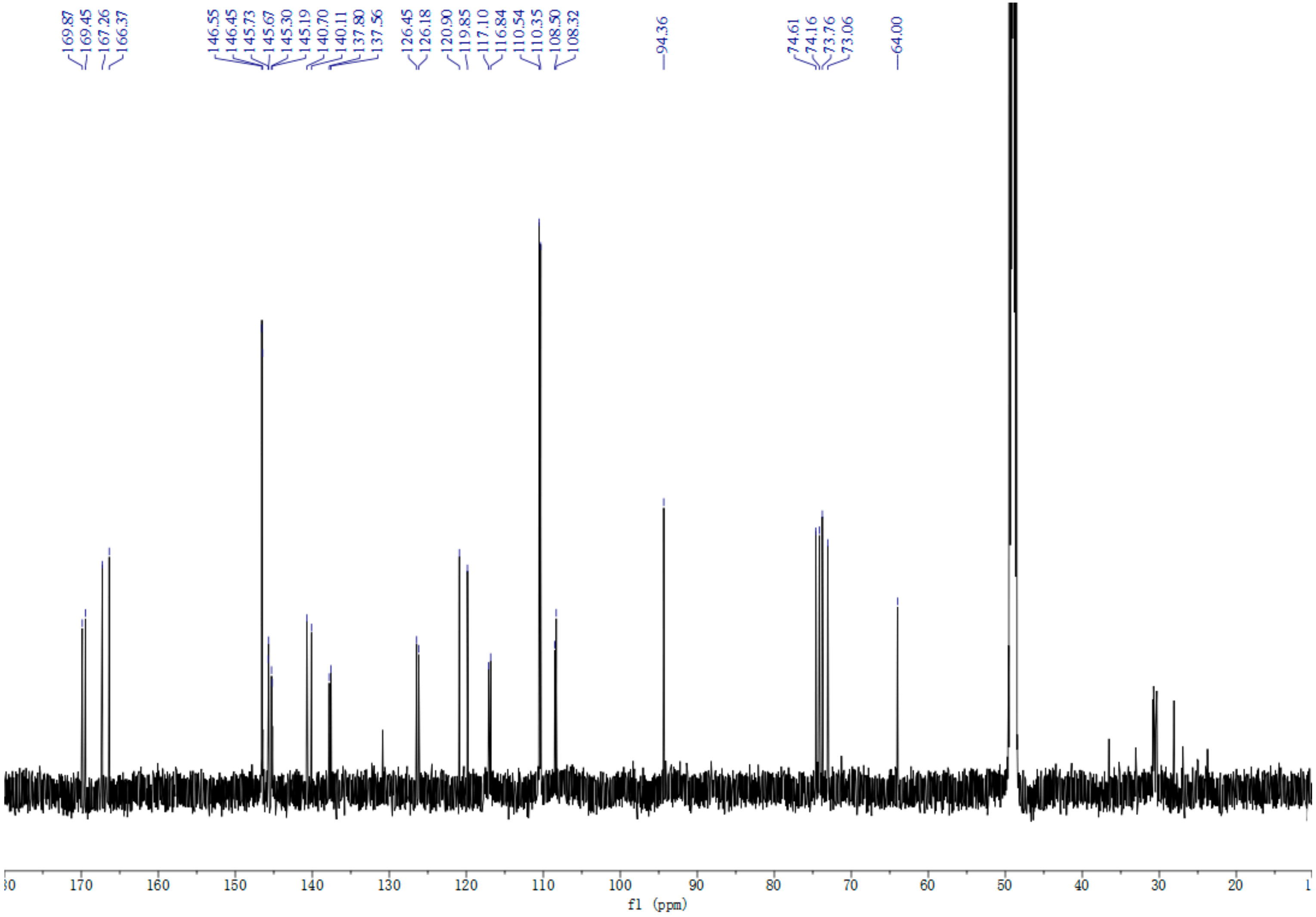

Figure S19. ^{1}H NMR spectrum of compound **6** in CD_3OD

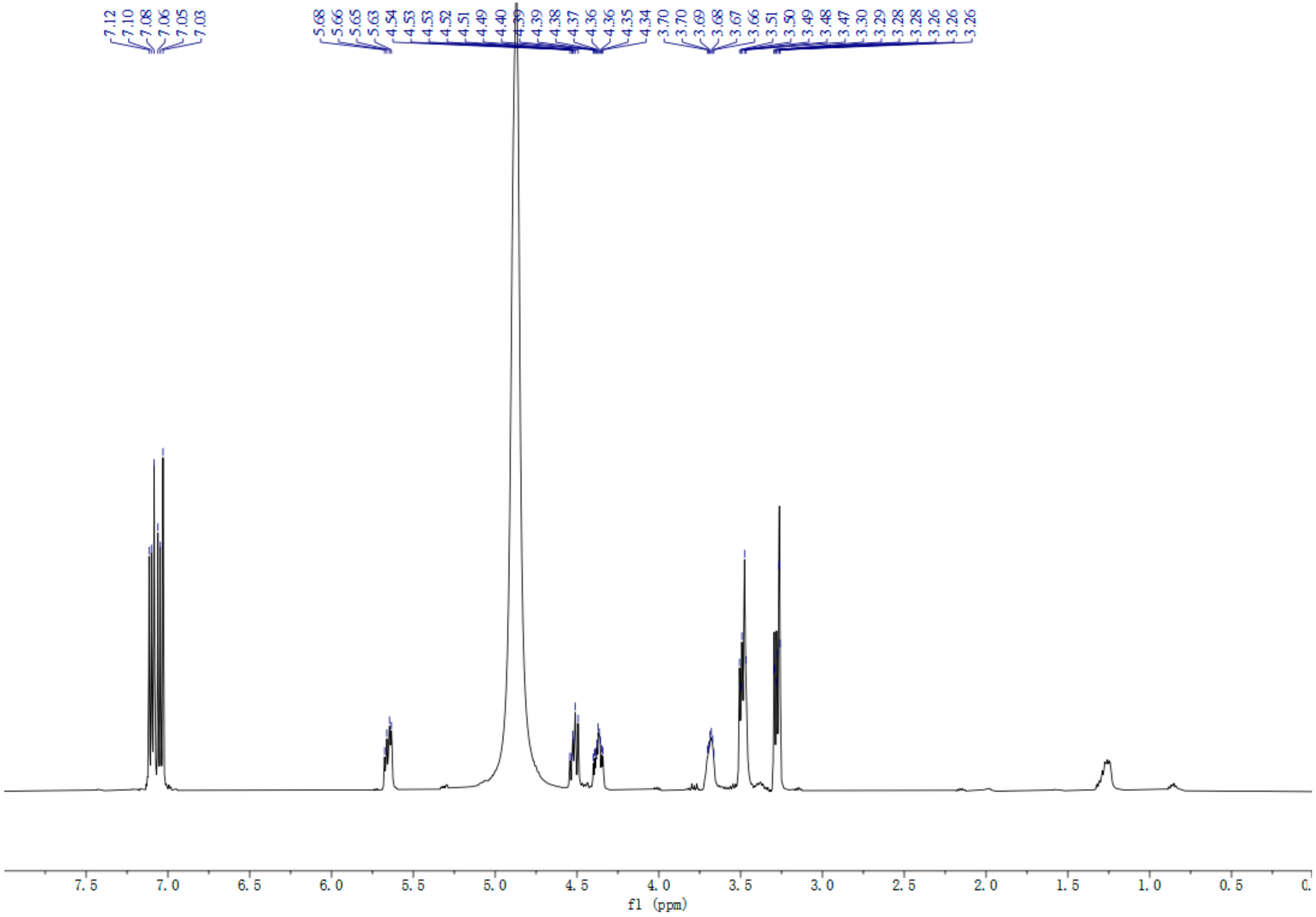

Figure S20. ^{13}C NMR spectrum of compound **6** in CD_3OD

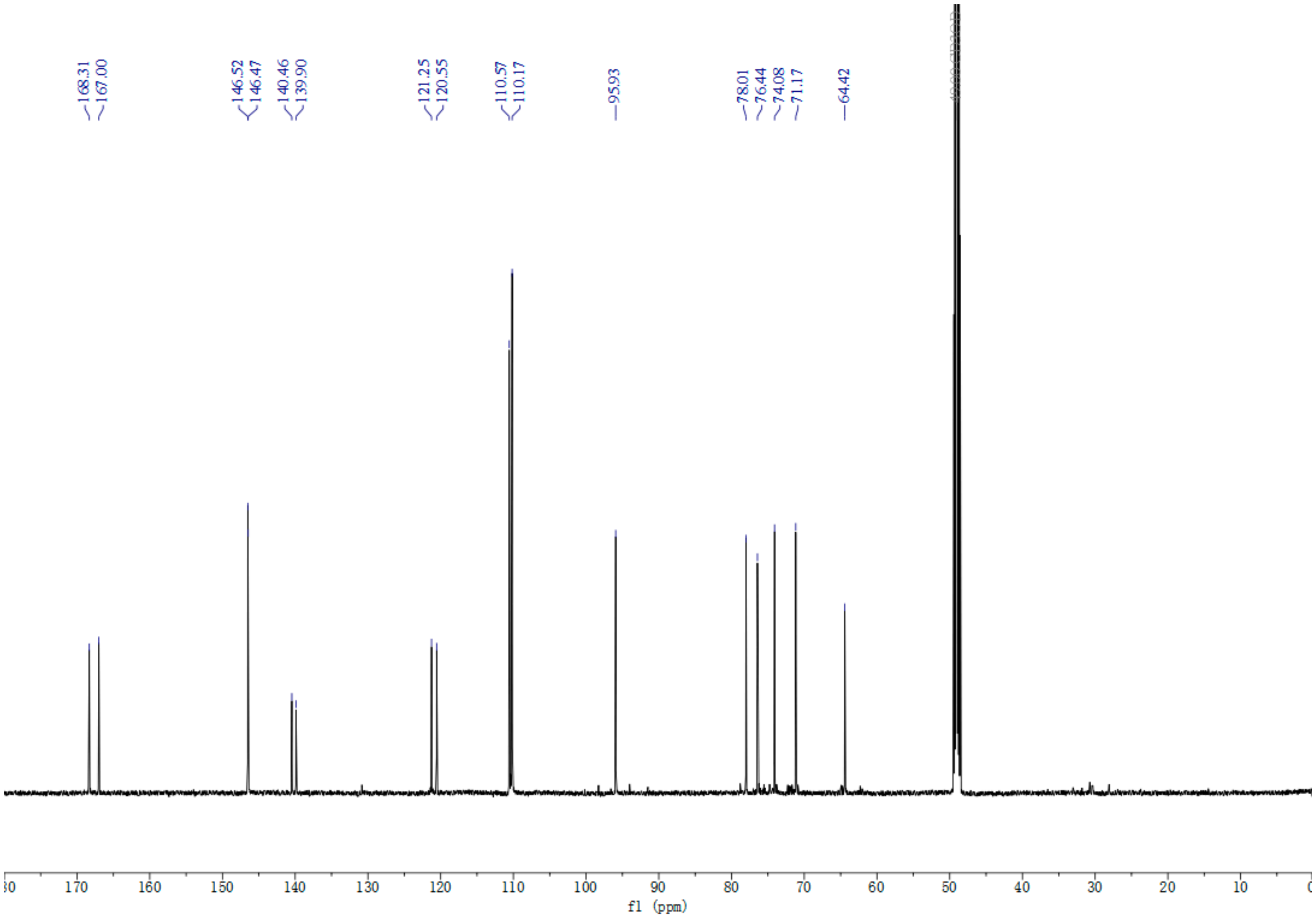

Figure S21. ^{1}H NMR spectrum of compound **7** in CD_3OD

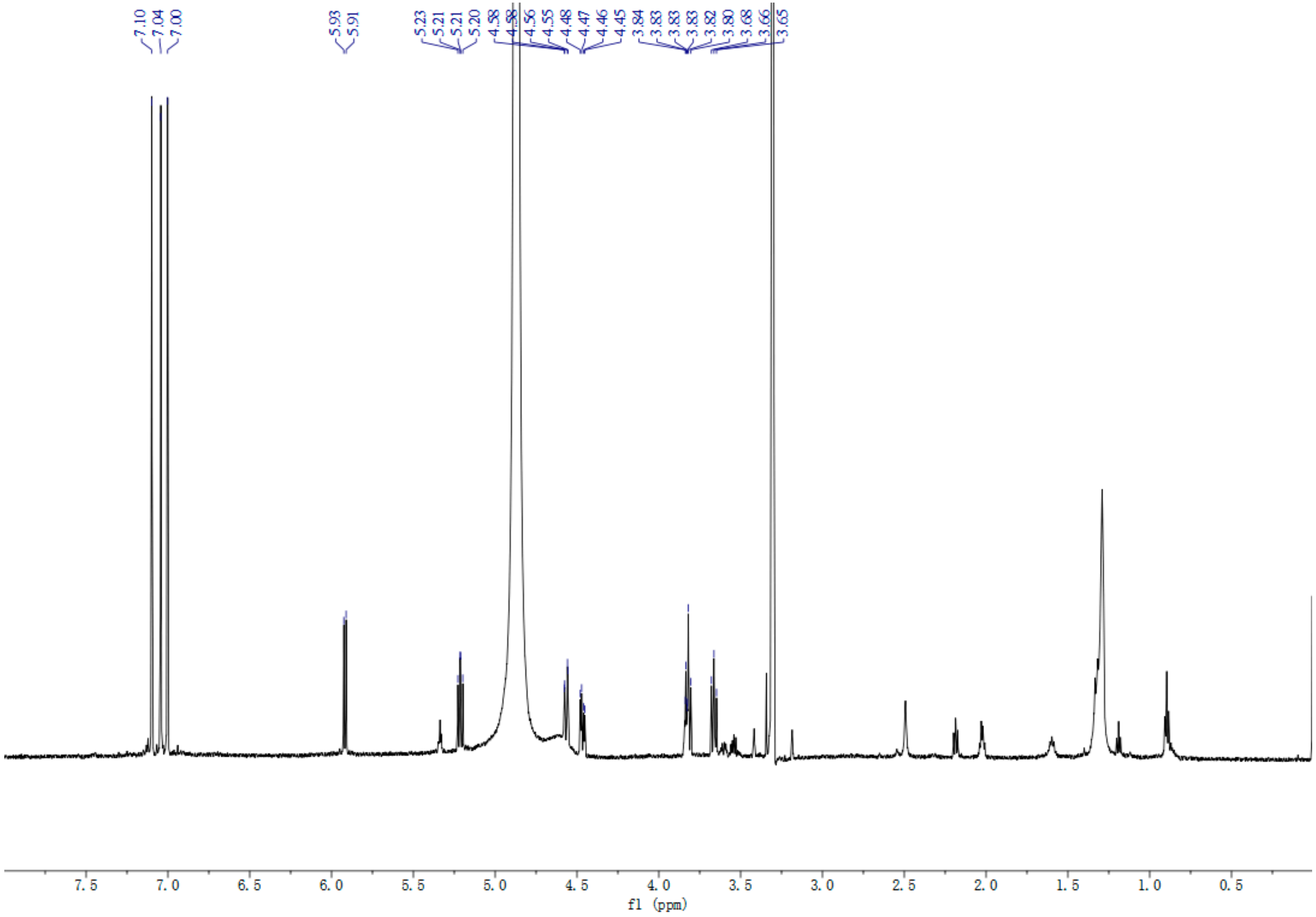

Figure S22. ^{13}C NMR spectrum of compound **7** in CD_3OD

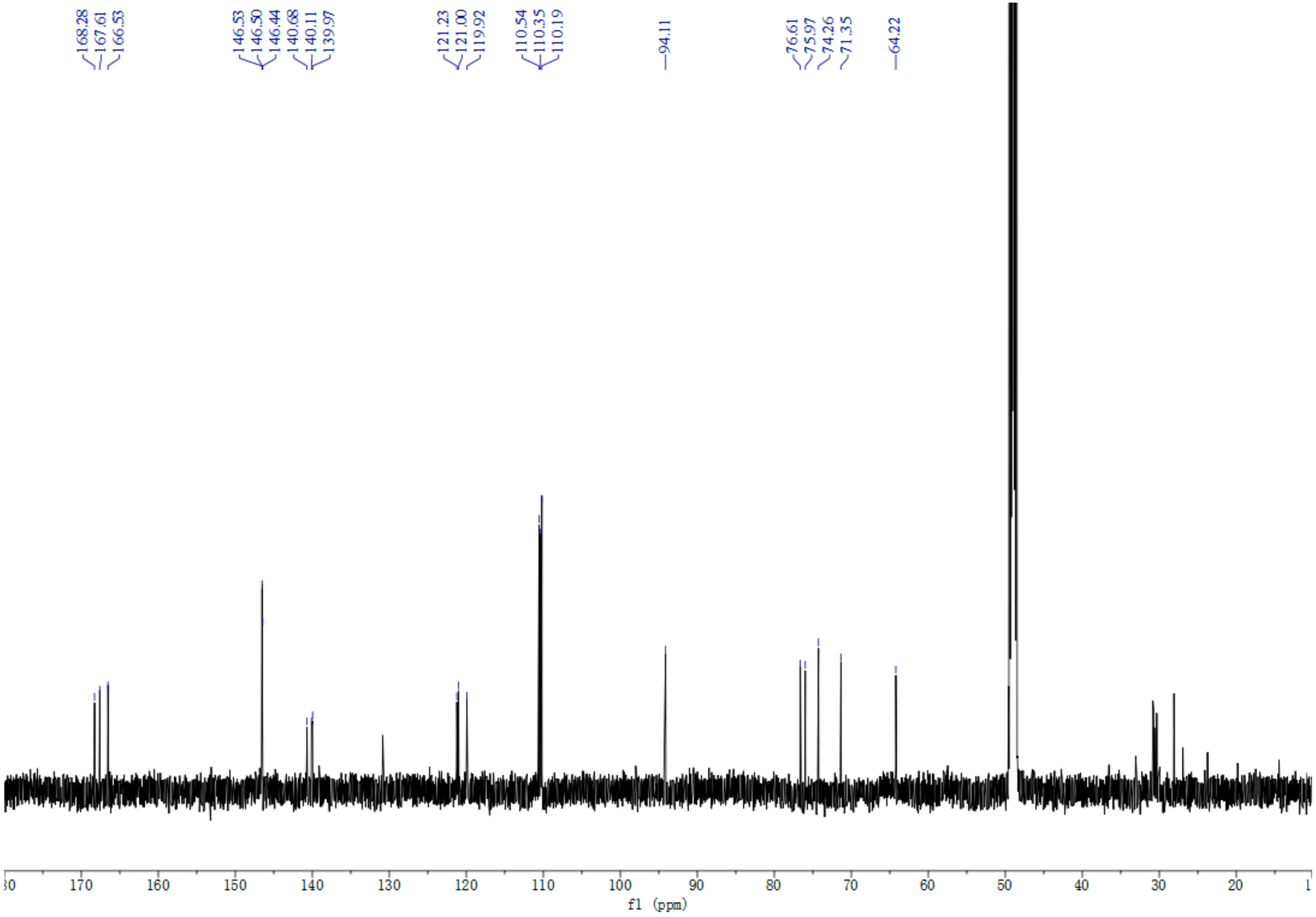

Figure S23. ^{1}H NMR spectrum of compound **8** in CD_3OD

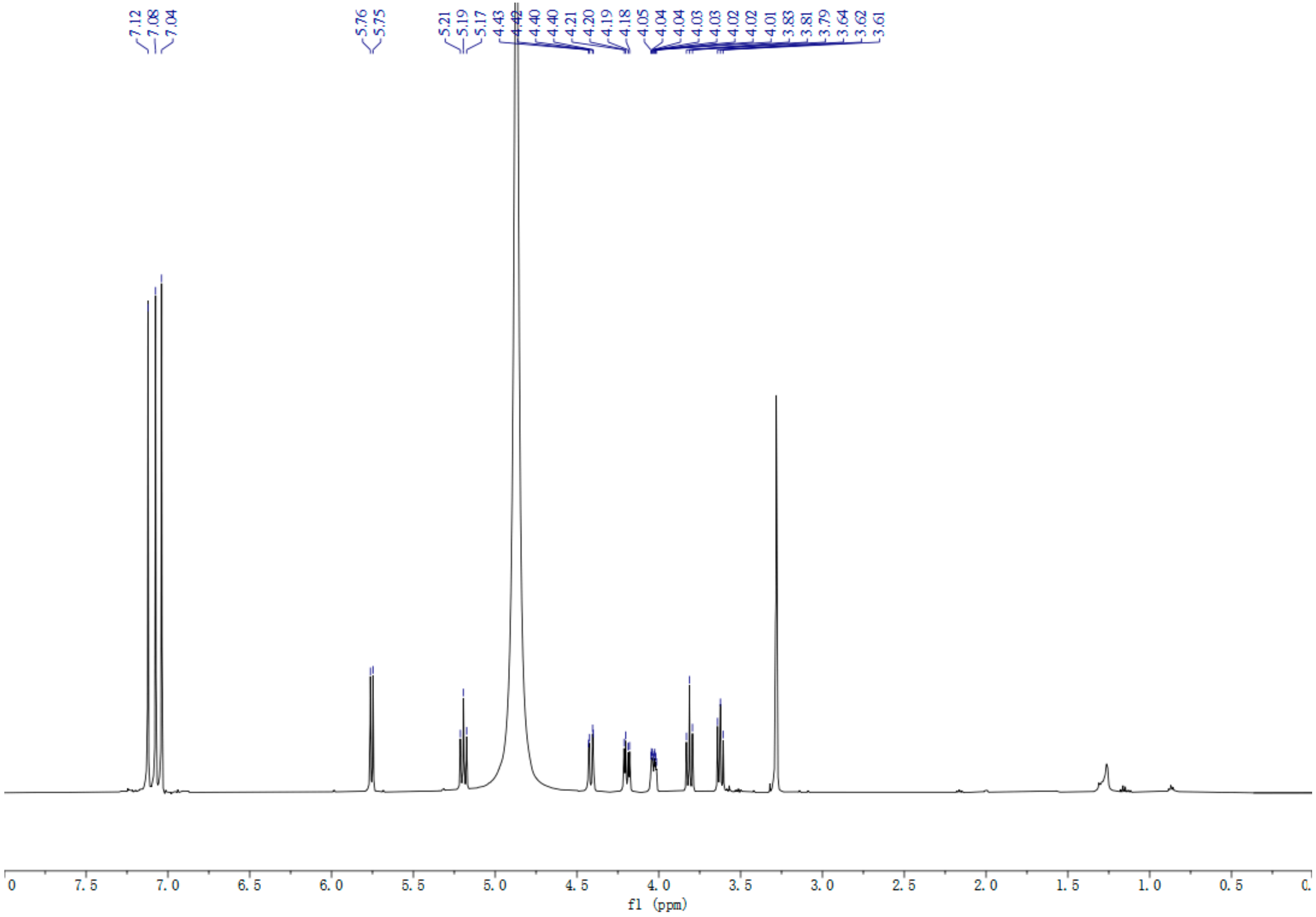

Figure S24. ^{13}C NMR spectrum of compound **8** in CD_3OD

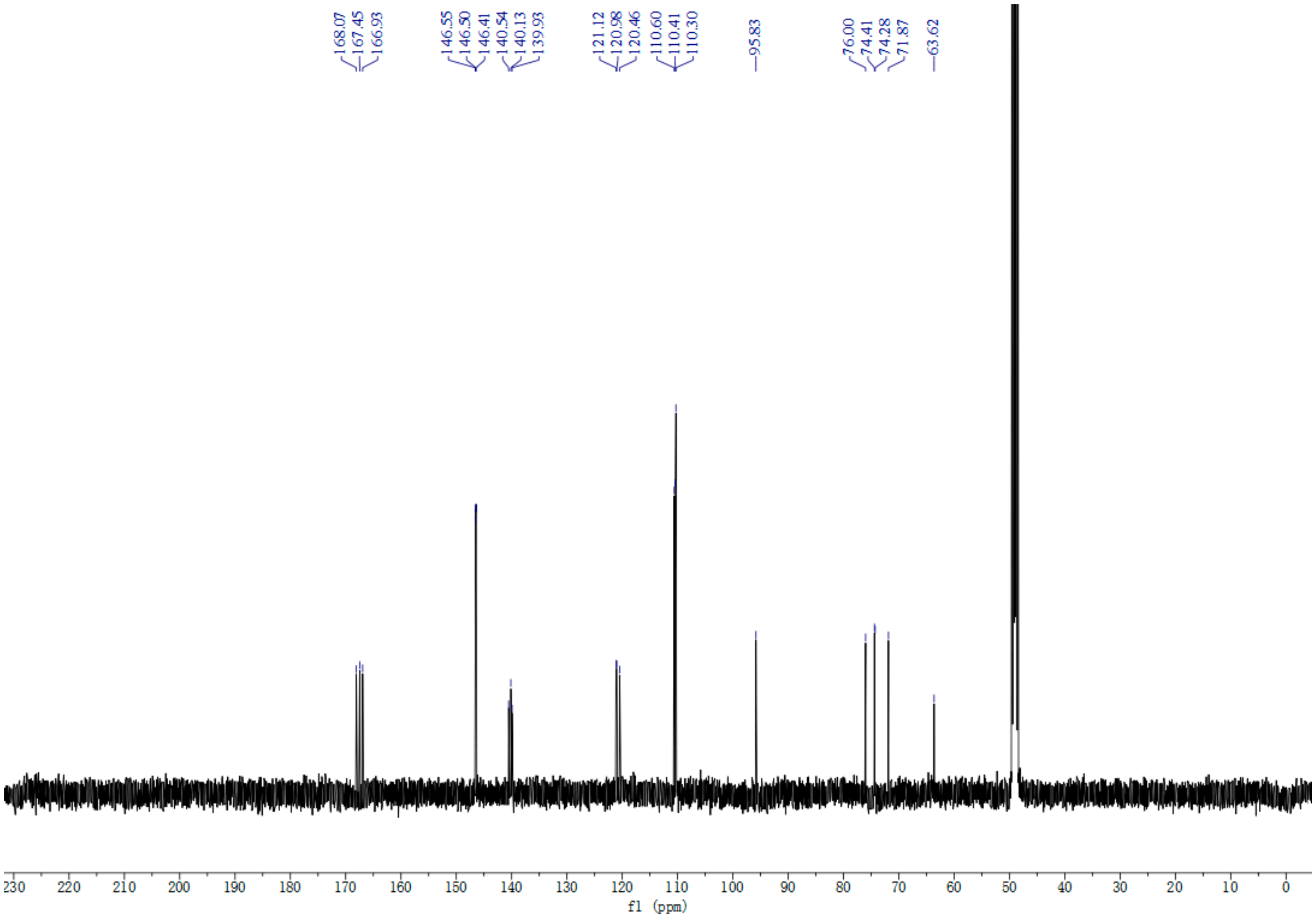

Supplement: Supplementary file 1 [file foods-12-02512-s001.zip › foods-2450752-supplementary.pdf]
